# *De novo* genome assembly of the endangered *Acer yangbiense*, a plant species with extremely small populations endemic to Yunnan Province, China

**DOI:** 10.1093/gigascience/giz085

**Published:** 2019-07-15

**Authors:** Jing Yang, Hafiz Muhammad Wariss, Lidan Tao, Rengang Zhang, Quanzheng Yun, Peter Hollingsworth, Zhiling Dao, Guifen Luo, Huijun Guo, Yongpeng Ma, Weibang Sun

**Affiliations:** 1 Yunnan Key Laboratory for Integrative Conservation of Plant Species with Extremely Small Populations, Kunming Institute of Botany, Chinese Academy of Sciences, Kunming, 650201, China; 2 Key Laboratory for Plant Diversity and Biogeography of East Asia, Kunming Institute of Botany, Chinese Academy of Sciences, Kunming, 650201, China; 3 University of Chinese Academy of Sciences, Beijing, 100049, China; 4 Beijing Ori-Gene Science and Technology Co. Ltd, Beijing, 102206, China; 5 Royal Botanic Garden Edinburgh, 20a Inverleith Row, Edinburgh, UK; 6 Southwest Forestry University, Kunming, 650224, Yunnan, China; 7 Kunming Botanical Garden, Kunming Institute of Botany, Chinese Academy of Sciences, Kunming, 650201, China

**Keywords:** *Acer yangbiense*, PSESP, PacBio sequencing, genome assembly, genome annotation

## Abstract

**Background:**

*Acer yangbiense* is a newly described critically endangered endemic maple tree confined to Yangbi County in Yunnan Province in Southwest China. It was included in a programme for rescuing the most threatened species in China, focusing on “plant species with extremely small populations (PSESP)”.

**Findings:**

We generated 64, 94, and 110 Gb of raw DNA sequences and obtained a chromosome-level genome assembly of *A. yangbiense* through a combination of Pacific Biosciences Single-molecule Real-time, Illumina HiSeq X, and Hi-C mapping, respectively. The final genome assembly is ∼666 Mb, with 13 chromosomes covering ∼97% of the genome and scaffold N50 sizes of 45 Mb. Further, BUSCO analysis recovered 95.5% complete BUSCO genes. The total number of repetitive elements account for 68.0% of the *A. yangbiense* genome. Genome annotation generated 28,320 protein-coding genes, assisted by a combination of prediction and transcriptome sequencing. In addition, a nearly 1:1 orthology ratio of dot plots of longer syntenic blocks revealed a similar evolutionary history between *A. yangbiense* and grape, indicating that the genome has not undergone a whole-genome duplication event after the core eudicot common hexaploidization.

**Conclusion:**

Here, we report a high-quality *de novo* genome assembly of *A. yangbiense*, the first genome for the genus *Acer* and the family Aceraceae. This will provide fundamental conservation genomics resources, as well as representing a new high-quality reference genome for the economically important *Acer* lineage and the wider order of Sapindales.

## Data Description

### Background information

The genus *Acer* L., commonly known as maple, is one of the most important genus of trees and shrubs in the Northern Hemisphere [1–5]. *Acer* exhibits a classical pattern of biogeographic disjunction across Europe, Northern Africa, Asia, and North America, with the greatest species richness in Eastern Asia [2, 4, 6–12]. It is a wide-ranging genus comprising up to 129 species worldwide with maximum diversity in China, where ∼99 (61 endemic, 3 introduced) species are recognized [13]. The base chromosome number of *Acer* is x = 13, and cytological investigation indicates a range of ploidy levels including diploids, tetraploids, hexaploids, octoploids, and aneuploids [14]. *Acer* has long been of interest to botanists for its remarkable diversity, especially of leaves, fruits, and bark, and for its intercontinental disjunct distribution [7]. The colorful foliage of maples is a charismatic landscape feature, with vivid hues of red, yellow, and orange in the autumn. In addition to being ornamental, many species are sources of commercial products, such as maple syrup, furniture, and timber [15]. Maple has been found to contain a large number of phytochemicals that have antioxidant, antitumor, and anti-inflammatory activities [15–20].

**Figure 1:**
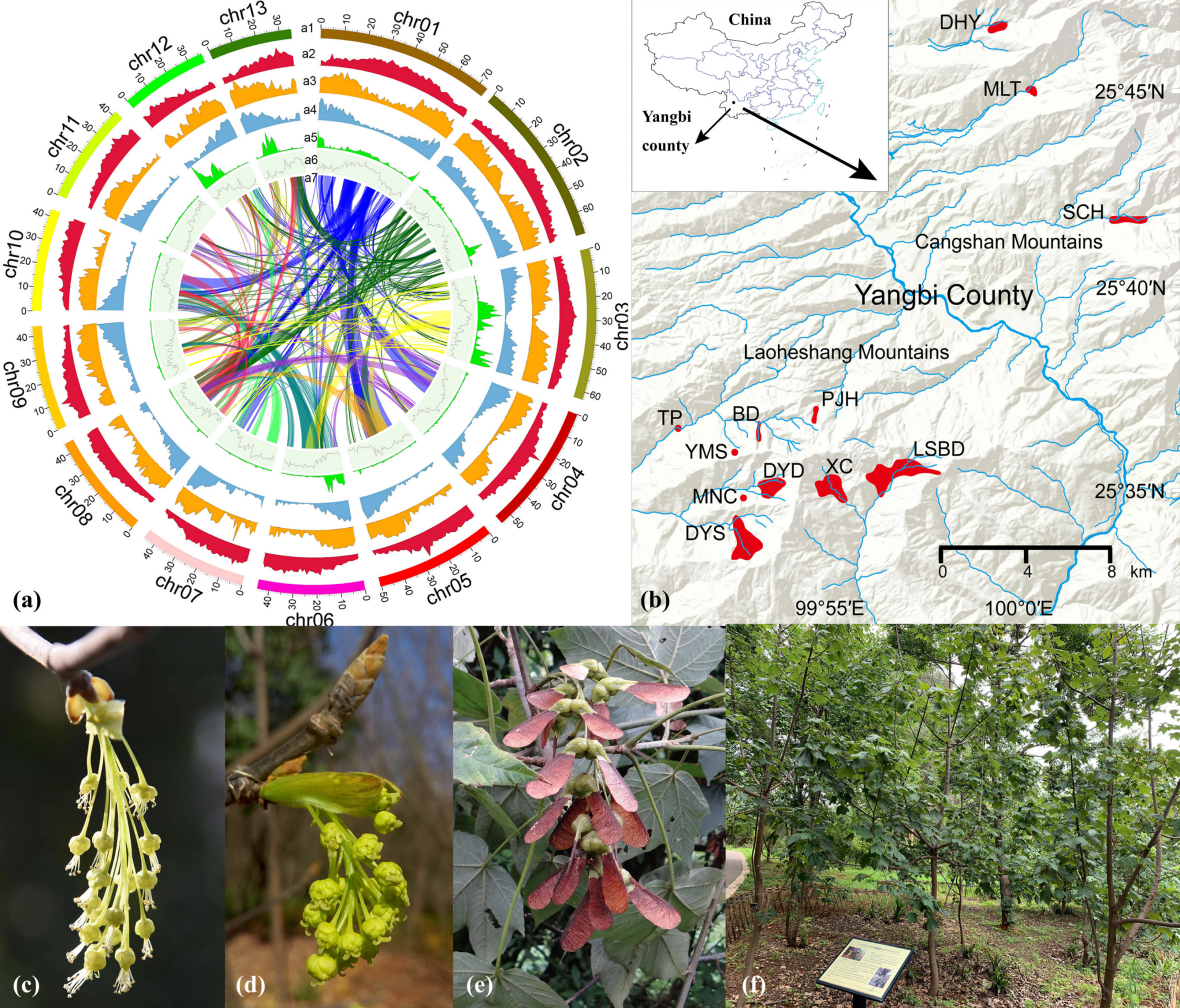
Images of *Acer yangbiense* chromosome assembly, distribution range, flowers, fruits, and *ex situ* conserved tree. **(a)** Genome features across 13 chromosomes. The tracks represent 13 assembled chromosomes (a1), Class I TE (LTRs, long interspersed nuclear elements, and short interspersed nuclear elements) density (a2), Class II TE (DNA and Heliron) density (a3), gene (messenger RNA) density (a4), heterozygous (single-nucleotide polymorphisms, insertions, and deletions) density (a5), GC content (a6), and genome rearrangement events of colinear blocks (a7). **(b)** Red-shaded regions denote distribution range of *A. yangbiense* in Yangbi county. Location codes: BD: Badahe; DHY: Dahuayuan; DYD: Diaoyudao; DYS: Dayingshan; LSBD: Luosibaidi; MLT: Malutang; MNC: Maoniuchang; PJH: Panjiahe; SCH: Sanchahe; TP: Taiping; XC: Xincun; YMS: Yangmeishu). **(c)** Staminate inflorescence. **(d)** Pistillate inflorescence. **(e)** Fruits. **(f)***ex-situ* conserved tree.

*Acer yangbiense* Y. S. Chen & Q. E. Yang (Aceraceae, NCBI:txid1000413) is a newly described Chinese maple species (Fig. 1) [21]. It has a restricted distribution range of 2,200–2,500 m altitudes in the western valley of Cangshan Mountain, Yunnan Province, China. This species is facing a very high risk of extinction because of its small population size, poor reproduction, and habitat degradation [22]. The species was categorized as critically endangered (CR) by Gibbs and Chen in 2009 [23], and as only 5 individuals were recorded based on Qin et al. (2017) [24–26] in the first decade after its description. In 2016, further survey work recovered a total of 577 individuals from 12 localities [27]. This is the most accurate available population estimate of *A. yangbiense*.

*A. yangbiense* is classified as a “plant species with extremely small populations” (PSESP) by the Chinese government and included in the PSESP rescue plan [28, 29]. The concept of PSESP emphasizes species that face high risk of extinction, characterized by small remaining populations in restricted habitats and being subjected to severe human disturbance [28, 30]. It is targeted at species with <5,000 mature individuals in total and <500 mature individuals in each isolated population [31]. Genetic studies done by Yang et al. (2015) suggested that *A. yangbiense* was not genetically depauperate, but further parentage analysis indicated a high selfing rate in seedlings of *A. yangbiense* [25]. The current threatened status of *A. yangbiense* serves to emphasize that an effective conservation strategy is urgently required.

The generation of plant genome sequences and assemblies allows detailed insights into the evolutionary history of species and provides information to support sustainable conservation [32]. Here, we present a high-quality genome assembly of *A. yangbiense* as a valuable resource and reference for future population genomic studies. The availability of a fully sequenced and annotated genome is essential to resolve fundamental questions about *A. yangbiense* diversification and provide new insights into its demographic history, with important implications for future conservation efforts.

### Plant material

Fresh young leaves were collected from *ex situ* conserved *A. yangbiense* at the Kunming Botanical Garden (KBG) of the Kunming Institute of Botany, Chinese Academy of Sciences. This tree was grown from seed in 2009, from seeds originally collected from Malutang, Yangbi County, Dali, Yunnan (Fig. 1) (25.7489 N latitude, 100.0064 E longitude, 2,474 m elevation). For genome library preparation, only leaf tissues were used; for transcriptome sequencing, samples were obtained from 5 different tissues: leaf buds, young leaves, young stems, roots, and fruits from healthy individuals planted in KBG in June and July 2018, respectively. All samples were collected with permission from KBG. For RNA samples, tissues were immediately transferred into liquid nitrogen and stored in dry ice until RNA extraction; for DNA samples, tissues were immediately stored in dry ice until DNA extraction.

### PacBio SMRT sequencing

Genomic DNA with high quality and high molecular weight was extracted from fresh leaves using a cetyl trimethylammonium bromide protocol [33]. Libraries for single-molecule real-time (SMRT) Pacific Biosciences (PacBio) genome sequencing were constructed following the standard protocols of PacBio at Beijing Ori-Gene Science and Technology Co., Ltd (Beijing, China). Briefly, 50 μg of high-quality genomic DNA was sheared to ∼20 kb targeted size, followed by damage repair and end repair, blunt-end adapter ligation, and size selection. Finally, the libraries were sequenced on the PacBio Sequel platforms using S/P2-C2 sequencing chemistry (10 SMRT cells). A total of 6.3 million PacBio reads with ∼64 Gb sequencing data were generated, with an average read length of 10 kb. The longest read was 93 kb, and N50 was 16.8 kb (Supplementary Table S1).

### Illumina sequencing

The Illumina libraries were constructed according to the standard manufacturer's PCR-free protocol (Illumina). Short-insert libraries of 300–500 bp were prepared using 2 μg of whole-genomic DNA for Illumina sequencing. All the libraries were sequenced on Illumina HiSeq X platform with paired-end (PE) sequencing strategy. In total, 3 PCR-free libraries were generated, and Fastp v0.19.3 (fastp, RRID:SCR_016962) [34] was used to filter out low-quality reads and adapter sequences. A total of 624.149 million raw reads was generated. This produced ∼94.246 Gb (∼140× the assembled genome) of raw sequencing data, with an average cleaned read length of 148.5 bp (Supplementary Table S2).

### Hi-C sequencing

The Hi-C library was prepared by Beijing Ori-Gene Science and Technology Co., Ltd (Beijing, China), with the standard procedure described as follows. A total of 700 ng of high molecular weight genomic DNA was cross-linked *in situ*, extracted, and then digested with a restriction enzyme. The sticky ends of the digested fragments were biotinylated, diluted, and then ligated to each other randomly. Biotinylated DNA fragments were enriched and sheared to a fragment size of 300–500 bp again for preparing the sequencing library, which was sequenced on a HiSeq X Ten platform (Illumina). A total of 740 million reads with ∼110 Gb sequencing data were generated (∼170× the assembled genome) with an average read length of 149.8 bp (Supplementary Table S3). During preprocessing of the Illumina data, Fastp v0.19.3 [34] was used to remove the short reads, low-quality, and adapter sequences.

### Estimation of genome size, heterozygosity, and repeat content

Three short fragment libraries were constructed by PCR-free method, and the whole-genome shotgun (WGS) short reads were generated using an Illumina HiSeq X Ten machine, which were filtered and corrected with Fastp v0.19.3 [34]. The genome size of *A. yangbiense* was estimated by the *k*-mer method [35] using sequencing data from the Illumina DNA library. First, Jellyfish v2 (Jellyfish, RRID:SCR_005491) [35] was used to count the occurrence of *k*-mers based on the processed data. Finally, gce v1.0.0 [36] was used to estimate the overall characteristics of the genome, such as genome size, repeat contents, and level of heterozygosity. In this study, 67,781,536,308 *k*-mers were generated, and the peak *k*-mer depth was 111 (Supplementary Fig. S1). The genome size was estimated to be ∼640 Mb, and repeat and heterozygosity rates were estimated to be 68.75% and 0.19%, respectively, based on *k*-mer individuals (Supplementary Table S4).

### *De novo* assembly and chromosome construction

The *de novo* genome assembly was performed on full PacBio long reads using different assembly strategies to obtain a better genome assembly. Primary assembly v0.1 was generated from PacBio long reads by Canu v1.7 (Canu, RRID:SCR_015880) [37], assembly v0.2 by SMARTdenovo v1.0 [38], assembly v0.3 by Wtdbg v1.2.8 (WTDBG, RRID:SCR_017225) [39], assembly v0.4 after correction by Canu v1.7 [37] and SMARTdenovo v1.0 [38], assembly v0.5 after corrected by Canu v1.0 [37] and Wtdbg v1.2.8 [39], assembly v0.6 after corrected and trimmed by Canu v1.7 [37] and SMARTdenovo v1.0 [38], and assembly v0.7 after corrected and trimmed by Canu v1.7 and Wtdbg v1.2.8 [39] (Supplementary Table S5). The assembly (v0.4) from SMARTdenovo v1.0 [38] after Canu v1.7 [39] correction was chosen as the optimal assembly for further polishing and scaffolding. In this selected primary assembly (v0.4), the assembled genome size was 666 Mb distributed across 880 contigs with N50 of 2.3 Mb, L50 of 84, and maximum contig length of 11.9 Mb (Supplementary Table S5). The draft assembly was first polished with Pilon v1.22 (Pilon, RRID:SCR_014731) [40] based on the high-quality Illumina sequencing reads and then piped into the Hi-C assembly workflow. Clean Hi-C reads were mapped to the draft assembly with Juicer (Juicer, RRID:SCR_017226) [41], and then a candidate chromosome-length assembly was generated automatically using the 3d-DNA pipeline to correct mis-joins, order, orient, and anchor contigs from the draft assembly [42]. Manual review and refinement of the candidate assembly was performed in Juicebox Assembly Tools (JBAT) [43] for quality control and interactive correction. To reduce the influence of interactions of chromosomes and to further improve the chromosome-scale assembly, each chromosome was re-scaffolded with 3d-DNA [42] separately, and then manually refined with Juicebox [44]. With the modified 3d-DNA and JBAT workflow, 13 chromosomes (646,206,981 bp, ∼97.04%) were anchored with only 265 contigs (18,721,930 bp) unplaced. Finally, after gap filling with LR_GapCloser v1.1 (GapCloser, RRID:SCR_015026) [45] (based on PacBio long reads, running for 2 rounds), Pilon v1.22 (Pilon, RRID:SCR_014731) was used to polish the assembly (based on Illumina reads, running for 5 rounds), and Redundans v0.13 [46] was used to remove putative haplotigs, to obtain the final genome assembly (v1.1) (Supplementary Table S5). In this final genome assembly v1.1 (Supplementary Table S5), we achieved an assembled genome size of 666 Mb characterized by 562 contigs and 280 scaffolds (with contig N50 of 5.5 Mb and scaffold N50 of 45 Mb) (Table 1 and Supplementary Table S5).

**Table 1:** *A. yangbiense* final genome assembly statistics

	Contig		Scaffold	
Characteristic	Size (bp)	Number	Size (bp)	Number
**Total size**	665,887,899 bp	562		280
**N10**	10,447,168 bp	5	73,781,861 bp	1
**N50**	5,479,097 bp	39	44,917,698 bp	6
**N90**	835,514 bp	154	36,383,401 bp	12
**Maximum**	17,438,070 bp		73,781,861 bp	
**Minimum**	7,640 bp		7,640 bp	
**Mean**	1,184,817 bp		2,378,171 bp	
**Median**	137,049 bp		50,985 bp	
**Gap**				282
**GC content**	35.96%			

### Assessment of genome assembly

We evaluated the level of genome completeness of the final genome assembly using BUSCO (RRID:SCR_015008) [47] and the LTR Assembly Index (LAI) [48]. BUSCO analysis showed that 95.5% (90.8% complete and single-copy genes and 4.7% complete and duplicated genes) and 2.2% of the 1,440 expected embryophytic genes were identified as complete and fragmented genes, respectively (Table 2). In addition, a relatively high LAI score = 12.21 (categorized as reference level when 10 ≤ LAI ≤ 20) showed that the assembly yielded high sequence continuity [48], agreeing with the BUSCO completeness. (Supplementary Table S5). The overall mapping rate of transcriptome data was 95.0% by HiSat2 v2.1.3 (HISAT2, RRID:SCR_015530) [49], showing good completeness of the assembly. The mapping of the whole Illumina short reads by BWA v0.7.17-r1188 (BWA, RRID:SCR_010910) [50] was 99.4%, which means almost all sequencing data were represented (covering 98.4% of the total genome length, among which, 97.9% with a coverage depth ≥5×, 97.7% with a coverage depth ≥10×, 97.4% with a coverage depth ≥20×, showing high coverage). Meanwhile, mapping of PacBio reads and bases by minimap2 v2.11-r797 [51] was 76.5% and 94.3%, respectively (covering 99.98% of the total length of genome, among which, 99.9% with a coverage depth ≥5×, 99.8% with a coverage depth ≥10×, and 99.4% with a coverage depth ≥20×). Both coverage rates of Illumina sequencing and PacBio sequencing were consistent and relatively high. The coverage depth distribution of the whole genome, as well as both gene regions of single-copy and duplicated BUSCO core genes, was plotted. The duplicated genes had the same depth distribution as the single-copy genes, indicating that the duplicated genes were not derived from unmerged haplotigs and thus there was almost no redundancy in the assembly (Supplementary Fig. S2). SAMtools (SAMtools, RRID:SCR_005227) [52] was used to detect variant sites. The heterozygosity rate was calculated by heterozygosity sites, and the error rate of single bases was calculated by homozygosity sites. The heterozygosity rate was ∼0.097%, while the error rate was ∼0.0037%. A guanine-cytosine (GC) depth analysis was conducted to assess potential contamination during sequencing and the coverage of the assembly, revealing that the genome had a mean GC content of 35.96% with no obvious GC bias (Supplementary Fig. S3). We searched all sequences of the genome assembly against the NCBI non-redundant nucleotide database with BLASTN to assess contamination, and the results suggested no potential contamination. When the Hi-C data were mapped to the final genome assembly using Juicer [41], the cluster results showed that there were 13 unambiguous chromosome scaffolds with no obvious chromosome assembly error (Supplementary Fig. S4).

**Table 2:** Summary of BUSCO evaluation of the gene prediction

Parameter	BUSCO groups (%)
**Complete BUSCOs**	1,375 (95.5)
**Complete and single-copy BUSCOs**	1,308 (90.8)
**Complete and duplicated BUSCOs**	67 (4.7)
**Fragmented BUSCOs**	29 (2.0)
**Missing BUSCOs**	36 (2.5)
**Total BUSCO groups searched**	1,440 (100)

### DNA repeats annotation

To *de novo* identify and classify repeat families in the genome assembly, the software package RepeatModeler v1.0.8 (RepeatModeler, RRID:SCR_015027) [53] was used with 2 complementary computational methods for *de novo* identifying repeats within the genome: RECON v1.08 and RepeatScout v1.0.5 (RepeatScout, RRID:SCR_014653) [54]. Then, using the output data file from RepeatModeler as a custom repeat library, RepeatMasker v4.0.7 (RepeatMasker, RRID:SCR_012954) [55] was used to screen for repeats within the assembled genome. In summary, repeat sequences were estimated to account for 68.0% (452.81 Mb) of the *A. yangbiense* assembly, among which 17.32% were uncharacterized repeats. Long terminal repeats (LTRs) were dominant (250.98 Mb, 37.7%), with Copia (179.64 Mb) and Gypsy (66.18 Mb), the most abundant subtypes, representing 26.98% and 9.94% of the genome assembly, respectively. The results of repeat annotations are summarized in Supplementary Table S6.

### Transcriptome assembly

Total RNA was extracted from the stem, roots, fruits, buds, and leaves using the Trizol reagent (Sangon Biotech, Shanghai) according to the manufacturer's instructions (Invitrogen). RNA quality was assessed on a Nanodrop-2000 spectrophotometer. The PE RNA sequencing libraries were prepared using the NEBNEXT Ultra RNA Library Prep Kit for Illumina, and 150 bp PE sequencing was performed on an Illumina HiSeq X Ten platform. A total of 252.03 million raw reads were generated (Supplementary Table S7). Using HiSat2 v2.1.0 [49], raw reads from RNA sequencing were aligned to the genome assembly. Then reference genome-guided transcriptome assemblies were constructed with StringTie v1.3.5 (StringTie, RRID:SCR_016323) [56] and Trinity v2.0.6 (Trinity, RRID:SCR_013048) [57], respectively. *De novo* assembly was generated using Trinity. After that, transcriptome assemblies were combined and further refined with CD-HIT v4.6 (CD-HIT, RRID:SCR_007105) [58]. In the end, a 138.40 Mb transcriptome with 82,766 unique transcripts was obtained as RNA sequencing evidence in genome annotation. The summary is provided in Supplementary Table S8.

### Genome annotation

The MAKER2 genome annotation pipeline [59] was used to predict protein-coding genes. After the repetitive sequences were masked, AUGUSTUS v3.3.1 (AUGUSTUS, RRID:SCR_008417) [60] was used for *ab initio* gene prediction with model training based on 1,248 single-copy orthologs, which were predicted by BUSCO [47] from the genome assembly. Then, for evidence-based gene prediction, transcripts from RNA sequencing were aligned to the repeat-masked reference genome assembly with BLASTN (BLASTN, RRID:SCR_001598) and TBLASTX (TBLASTX, RRID:SCR_011823) from BLAST v2.2.28+ (NCBI BLAST, RRID:SCR_004870) [61]; protein sequences from *Arabidopsis thaliana* and *Dimocarpus longan* were aligned to the repeat-masked reference genome assembly with BLASTX (BLASTX, RRID:SCR_001653). After optimization with Exonerate v2.2.0 (Exonerate, RRID:SCR_016088) [62], MAKER package v2.31.9 (MAKER, RRID:SCR_005309) [59] was used to prepare gene model predictions. AED (Annotation Edit Distance) scores were generated for each of the predicted genes as part of the MAKER pipeline, in order to assess the quality of gene prediction. Non-coding RNAs in the genome were identified by searching from the Rfam database [63]. Gene sets were integrated into a non-redundant gene annotation, and its completeness was checked using BUSCO (the 1,440 single-copy orthologs from the embryophyta_odb9 database) [47].

From the assembled genome of *A. yangbiense* a total of 30,418 genes were annotated. Besides, 28,320 protein-coding genes were acquired; 25,572 of these had an AED < 0.5 and a mean of 5.36 exons per gene. The mean lengths of gene region, transcript, and coding DNA sequence were 3,880, 1,455, and 1,308 bp, respectively (Supplementary Table S9). With regard to noncoding RNA, 734 noncoding RNA, 248 ribosomal RNA, and 1,116 transfer RNA sequences were identified by Rfam (Rfam, RRID:SCR_007891), RNAMMER (RNAmmer, RRID:SCR_017075) [64], and tRNAScan-SE (tRNAscan-SE, RRID:SCR_010835) [65], respectively.

Gene function annotation was assigned on the basis of sequence and domain conservation. For assignment based on sequence conservation, a BLAT (E-value threshold of 1e−5) (BLAT, RRID:SCR_011919) [66] search of the peptide sequences from several protein databases was performed, such as Swiss-Prot [67, 68], TrEMBL [67, 69], NR [70], Pfam [71], and eggnog [72]. For assignment based on domain conservation, InterProScan (InterProScan, RRID:SCR_005829) [73] was used to examine motifs and domains by matching against public databases, such as ProDom [74], PRINTS [75], Pfam, SMART [76], PANTHER [77], and PROSITE [78]. The highest proportions of annotation were 92.60% (database NR) by BLAT and 92.31% (database PANTHER) by InterProScan, and the unannotated proportions were 7.30% and 1.74%, respectively (Supplementary Table S10).

### Identification of orthologous genes and phylogenetic tree construction

OrthoMCL v2.0.9 (Ortholog Groups of Protein Sequences, RRID:SCR_007839) [79] was used to identify orthologous and paralogous gene clusters in the assembled genomes of *A. yangbiense* and 14 related plant species (Supplementary Table S11), including *Arabidopsis thaliana* [80], *Theobroma cacao* [81], *Citrus grandis* [82], *Populus trichocarpa* [83], *Eucalyptus grandis* [84], *Vitis vinifera* [85, 86], *Coffea canephora* [87], *Beta vulgaris* [88], *Carica papaya* [89], *D. longan* [90], *Fragaria vesca* [91], *Medicago truncatula* [92], *Sclerocarya birrea* [93], and *Oryza sativa* [94]. Recommended settings were used for all-against-all BLASTP comparisons (BLAST+ v2.3.056) [61] and OrthoMCL [95] analysis.

A total of 29,892 OrthoMCL families including 379,261 genes were built on the basis of effective database sizes of all-vs-all BLASTP with an E-value of 10^−5^ and a Markov chain clustering default inflation parameter. In addition, 542 gene families with 1,793 genes were identified to be specific to the *A. yangbiense* genome when compared with the other 14 genomes (Supplementary Table S12). Furthermore, *A. yangbiense* and *D. longan* had the largest number of shared gene families (12,505) among the studied plants, supporting the closer relative relationships in the same family of Sapindaceae compared with other plant species (phylogeny of the angiosperms, APG IV) ([96] accessed 22 January 2019).

Phylogenetic analysis was performed using 854 orthologous protein-coding single-copy genes among the 15 genomes found by OrthoMCL [95]. These were then aligned with MUSCLE v3.8.31 (MUSCLE, RRID:SCR_011812) [97]. A maximum likelihood phylogenetic tree was then generated using the concatenated amino acid sequences in PhyML v3.0 with the default parameter (LG Model) [98]. The divergence time was estimated with r8s v1.81 [99] and calibrated against the divergence timing of Monocotyledoneae and Eudicotyledoneae (synchronously 135–130 million years ago [Mya]), of Pentapetalae (126–121 Mya), and Rosidae (123–115 Mya) [100]. The time-calibrated tree was further analysed together with these shared orthologous gene families among 15 plants by CAFE v4.0 [101], to detect expansion, contraction, and rapid evolution of those observed gene families.

**Figure 2:**
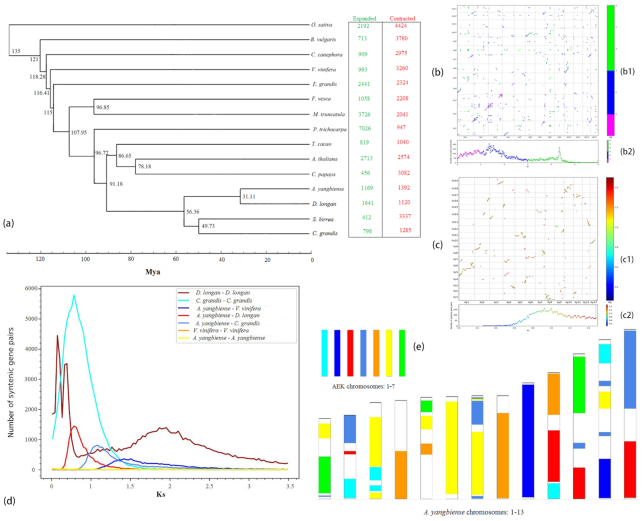
Genome evolution analysis of *A. yangbiense*. **(a)** Phylogenetic tree, divergence time, and profiles of gene families that underwent expansion or contraction. (b) Dot plots of syntenic blocks (b1) and corresponding Ks distribution histogram (b2) within *A. yangbiense*. (c) Dot plots of syntenic blocks (c1) and corresponding Ks distribution histogram (c2) between *A. yangbiense* and grape genome. **(d)** Synonymous substitution rate (Ks) distributions of syntenic blocks for *A. yangbiense* paralogs and orthologs with other eudicots are represented. **(e)** Comparison with ancestral eudicot karyotype (AEK) chromosomes reveals synteny. The syntenic AEK blocks are painted onto *A. yangbiense* chromosomes.

The phylogenetic analysis identified the closest relationship of *A. yangbiense* to *D. longan*, with the divergence time between them estimated at ∼31.11 Mya (Fig. 2a). Moreover the close relationship among Sapindaceae, Anacardiaceae (*S. birrea*), and Rutaceae (*C. grandis*) was confirmed, supporting the placement of the 3 families within the order of Sapindales in APG IV (Fig. 2a). Using CAFE v4.0 [101], a total of 1,169 gene families were detected that have expanded, while 1,392 gene families were found to have contracted in *A. yangbiense*. The expanded gene families were enriched for 209 significant (*q* < 0.05) GO terms of 3 categories, i.e., BP (Biological Process), CC (Cellular Component), and MF (Molecular Function) (Supplementary Table S13), and 5 KEGG pathways (Supplementary Table S14) significant at *q* < 0.05. Alternatively the contracted gene families were enriched for 334 GO terms of the aforementioned 3 categories (Supplementary Table S15) and 14 KEGG pathways (Supplementary Table S16) involving several aspects of secondary metabolism, at *q* < 0.05. Additionally, functional enrichment analysis of rapidly evolving gene families reveals 218 significant GO terms (Supplementary Table S17) and 17 KEGG pathways (Supplementary Table S18), both at *q* < 0.05.

### Genome evolution by synteny analysis

We performed synteny analysis of orthologous and paralogous genes previously identified by OrthoMCL [95] from *A. yangbiense* genomes, using MCScanX with default parameters, requiring ≥5 gene pairs per syntenic block [102]. The resulting dot plots were additionally used to assess characteristics of syntenic blocks by comparison within and between genomes (grape).

The Ks value was calculated to determine possible events of whole-genome duplication (WGD) and/or other duplications such as transposable elements (TE). First, protein sequences of those homologous colinear genes from *A. yangbiense* vs grape identified by MCScanX [102] were aligned against each other with MUSCLE (MUSCLE, RRID:SCR_011812) [97] to achieve the conserved protein sequences of each species, which were then converted into the corresponding codon alignments implemented in PAL2NAL [103]. Finally, Ks values were calculated by KaKs_Calculator [104] with YN model [105]. Based on the genome construction of the most recent ancestor of flowering plants, referred to as the ancestral eudicot karyotype (AEK) by Murat et al. [106], we compared the maple genome to AEK and then painted the syntenic AEK blocks onto *A. yangbiense* chromosomes.

A total of 999 colinear gene pairs on 139 colinear blocks were inferred within the *A. yangbiense* genome. There were 10,144 colinear gene pairs from 452 colinear blocks detected between *A. yangbiense* and grape (Supplementary Table S19). Dot plots of longer syntenic blocks between *A. yangbiense* and grape revealed a nearly 1:1 orthology ratio, indicating a similar evolution history to grape without undergoing a WGD event after the core eudicot common hexaploidization [107]. Synonymous substitution rate (Ks) distributions of syntenic blocks for *A. yangbiense* paralogs and orthologs with other eudicots also support the hypothesis of no recent WGD event (Fig. 2b–d). However, other than WGD, TE duplications might occur as the existence of short syntenic blocks in *A. yangbiense* (Fig. 2b). Furthermore, the genome painter image obtained by painting the syntenic AEK blocks onto *A. yangbiense* chromosomes illustrates that chromosomes 4, 6, 8, and 9 nearly exclusively contain the ancestral eudicot chromosome 2, 6, 5 without existence of inter-chromosomal segments (Fig. 2e). Such conserved gene content and order on these chromosomes in *A. yangbiense* could be due to the merged ancestral chromosome structures (e.g., multiple telomeres and centromeres on 1 chromosome) suppressing recombination and/or successive rearrangement, as was simultaneously inferred from the genome of *E. grandis* [84]. Last, we note that the genome of *A. yangbiense* has the potential to replace grape as the reference genome for studying recent WGD and chromosome evolution, especially for species within and/or close relatives to the order of Sapindales, due to high quality of genome assembly and no recent WGD, as well as less recombination of chromosomes in *A. yangbiense*.

## Conclusion

We have presented a *de novo* genome assembly of *A. yangbiense* using a combination of PacBio (SMRT), Illumina HiSeq X, and Hi-C approaches and achieved a high-quality sequence assembly. The *A. yangbiense* genome that we have sequenced, assembled, and annotated here is the first genome for the genus *Acer* and the family Aceraceae. This critically threatened species genome will facilitate the genome assembly and resequencing of additional species. It will be an essential resource for further investigations of the demography, adaptability, and conservation genetics of this endangered species. Likewise, the novel genome data generated in the present study will provide a valuable resource for studying WGD and chromosome evolution particularly in the Sapindales.

## Availability of supporting data and materials

The genome assembly, annotations, and other supporting data are available via the *GigaScience* database GigaDB [108]. This Whole Genome Shotgun project has been deposited at DDBJ/ENA/GenBank under the accession VAHF00000000. The version described in this paper is version VAHF01000000. The raw sequence data have been deposited in the Sequence Read Archive under NCBI BioProject ID PRJNA524417.

## Additional files

**Figure S1**. Frequency distribution of the 17-mer graph analysis used to estimate the size of the *A. yangbiense* genome.

**Figure S2**. Length distribution of PacBio subreads. Assessment of the distribution of genome reads (left) and BUSCO core region (right) coverage depth through PacBio-SMRT (lower) and Illumina sequencing data (upper).

**Figure S3**. Coverage depth of PacBio and Illumina sequencing data under different GCs. Assessment of the distribution of GC content and sequencing depth by PacBio-SMRT (left) and Illumina (right) under different GCs.

**Figure S4**. Hi-C map of final assembly of chromosomes. The distribution of links among chromosomes is exhibited by heat map based on HiCplotter. The heat map colors ranging from light yellow to dark red indicate the frequency of Hi-C interaction links from low to high (0–10).

**Table S1**. WGS-PacBio sequencing statistics

**Table S2**. WGS Illumina sequencing statistics

**Table S3**. Hi-C sequencing statistics

**Table S4**. *k*-mer survey statistics

**Table S5**. Statistics of all assemblies

**Table S6**. Repeat annotations of the *Acer yangbiense* genome assembly

**Table S7**. Summary of Illumina RNA sequencing data

**Table S8**. Summary of the transcriptome assemblies

**Table S9**. Gene annotation statistics of the *A. yangbiense* assembly

**Table S10**. Functional annotation of predicted genes in *A. yangbiense* genome

**Table S11**. Basic information with regard to genomes of 15 plants that were used for gene family analysis and phylogenetic tree construction

**Table S12**. Summary of the gene family analyses. Unique groups and genes, single-copy and duplicated groups and genes are summarized for the 15 plant genomes

**Table S13**. GO enrichment of expanded gene families. (A) “Category” is the Gene Ontology (GO) term ID; (B) “*P* value” is the over-represented *P*-value indicating that the observed frequency of a given term among analysed genes is equal to the expected frequency based on the null distribution; i.e., lower *P*-values indicate stronger evidence for overrepresentation; (C) “Q value” is the Benjamini-Hochberg adjusted *P*-value; (D) “numEPInCat” is the number of expanded gene families in the corresponding GO category; (E) “numInCat” is the number of detected gene families in the corresponding GO category; (F) “Term” is the GO term; (G) “Ontology” indicates which ontology the term comes from. Significant biological significance is at *q* < 0.05

**Table S14**. KEGG enrichment of expanded gene families. (A) “KO category” is the KEGG Orthology (KO) category ID; (B) “*P* value & Q value” have the same meaning as in Supplemental Table S13 (B) and (C); (D) “numEPInCat” is the number of expanded gene families in the corresponding KO category; (E) “numInCat” is the number of detected gene families in the corresponding KO category; (F) “Pathway” is the KEGG pathway; (G) “Class” indicates which KEGG class the pathway comes from. Significant biological significance is at *q*< 0.05

**Table S15**. GO enrichment of contracted gene families

**Table S16**. KEGG enrichment of contracted gene families

**Table S17**. GO enrichment of rapidly evolved gene families

**Table S18**. KEGG enrichment of rapidly evolved gene families

**Table S19**. Summary of colinear analysis within and between species

giz085_GIGA-D-19-00090_Original_Submission

giz085_GIGA-D-19-00090_Revision_1

giz085_Response_to_Reviewer_Comments_Original_Submission

giz085_Reviewer_1_Report_Original_Submission Tokurou Shimizu -- 4/20/2019 Reviewed

giz085_Reviewer_2_Report_Original_Submission John Hamilton -- 4/24/2019 Reviewed

giz085_Reviewer_3_Report_Original_Submission Meg Staton -- 4/29/2019 Reviewed

giz085_Supplemental_Files

## Abbreviations

AED: annotation edit distance; AEK: ancestral eudicot karyotype; APG: Angiosperm Phylogeny Group; BLAST: Basic Local Alignment Search Tool; bp: base pairs; BUSCO: Benchmarking Universal Single-Copy Orthologs; BWA: Burrows-Wheeler Aligner; Gb: gigabase pairs; GC: guanine-cytosine; gce: genome character estimation; GO: Gene Ontology; kb: kilobase pairs; KBG: Kunming Botanical Garden; KEGG: Kyoto Encyclopedia of Genes and Genomes; LAI: LTR Assembly Index; LTR: long terminal repeat; Mb: megabase pairs; Mya: million years ago; NCBI: National Center for Biotechnology Information; PacBio: Pacific Biosciences; PE: paired end; PSESP: plant species with extremely small populations; SMRT: Single-Molecule Real-Time; TE: transposable element; WGD: whole-genome duplication; WGS: whole-genome shotgun.

## Competing interests

The authors declare that they have no competing interests.

## Funding

This study was funded by NSFC (National Natural Science Foundation of China)-Yunnan Joint Fund (Grant No. U1302262), Science and Technology Basic Resources Investigation Program of China (Grant No. 2017FY100100), National Key R&D Program of China (Grant No. 2017YFC0505200), Yunnan Science and Technology Talents and Platform Program for Key Laboratory Construction (Grant No. 2018DG004), Yunnan Science and Technology Innovation Team Program for PSESP (Plant Species with Extremely Small Populations) Conservation and Utilization (Grant No. 2019HC015), and STS Program of the Chinese Academy of Sciences “Full Cover Conservation Project of Native Plants in Southwestern China” (KFJ-3W-No1).

## Authors' contributions

W.B.S. and Y.P.M. designed the study; L.D.T. and G.F.L. collected and prepared the materials; R.G.Z and Q.Z.Y conducted the experiments and data analysis. J.Y., H.M.W., and Y.P.M. wrote the manuscript; H.P., L.D.T., Z.L.D., H.J.G., and W.B.S. revised the manuscript. All authors read and approved the final draft.
