# Correction to: De novo genome assembly of the endangered Acer yangbiense, a plant species with extremely small populations endemic to Yunnan *De novo* genome assembly of the endangered *Acer yangbiense*, a plant species with extremely small populations endemic to Yunnan Province, China

**DOI:** 10.1093/gigascience/giz103

**Published:** 2019-09-11

**Authors:** Jing Yang, Hafiz Muhammad Wariss, Lidan Tao, Rengang Zhang, Quanzheng Yun, Peter Hollingsworth, Zhiling Dao, Guifen Luo, Huijun Guo, Yongpeng Ma, Weibang Sun

**Affiliations:** 1 Yunnan Key Laboratory for Integrative Conservation of Plant Species with Extremely Small Populations, Kunming Institute of Botany, Chinese Academy of Sciences, Kunming, 650201, China; 2 Key Laboratory for Plant Diversity and Biogeography of East Asia, Kunming Institute of Botany, Chinese Academy of Sciences, Kunming, 650201, China; 3 University of Chinese Academy of Sciences, Beijing, 100049, China; 4 Beijing Ori-Gene Science and Technology Co. Ltd, Beijing, 102206, China; 5 Royal Botanic Garden Edinburgh, 20a Inverleith Row, Edinburgh, UK; 6 Southwest Forestry University, Kunming, 650224, Yunnan, China; 7 Kunming Botanical Garden, Kunming Institute of Botany, Chinese Academy of Sciences, Kunming, 650201, China

*GigaScience*, Volume 8, Issue 7, July 2019, giz085, https://doi.org/10.1093/gigascience/giz085

The authors found an erroneous error in **Figure 1**, in that part of the territorial waters was missing in the map of China. The authors apologize that this error was not discovered before publishing. Therefore, to avoid misunderstandings and cause further problems, the authors have corrected this mistake. In the corrected Figure 1, the map of China was replaced with a correct one. The legend of Figure 1 will remain the same.

