# Supplementary material for: De novo genome assembly of the endangered Acer yangbiense, a plant species with extremely small populations endemic to Yunnan Province, China
Source: Gigascience. 2019 Jul 15;8(7):giz085. doi: 10.1093/gigascience/giz085 (PMC6629541; doi:10.1093/gigascience/giz085)
Supplement: giz085_GIGA-D-19-00090_Original_Submission [file giz085_giga-d-19-00090_original_submission.pdf]

# GigaScience

## De novo genome assembly of the endangered Acer yangbiense, a plant species with extremely small populations endemic to Yunnan of China

--Manuscript Draft--

| | |
|---|---|
| **Manuscript Number:** | GIGA-D-19-00090 |
| **Full Title:** | De novo genome assembly of the endangered Acer yangbiense, a plant species with extremely small populations endemic to Yunnan of China |
| **Article Type:** | Data Note |
| **Funding Information:** | Science and Technology Basic Resources Investigation Program of China (2017FY100100) — Prof. Wei Bang Sun<br>National Natural Science Foundation of China (U1302262) — Prof. Wei Bang Sun<br>Key Technologies Research and Development Program of China (2017YFC0505200) — Dr. Jing Yang<br>Key Laboratory Construction of Yunnan Science and Technology Talents and Platform Program (2018DG004) — Prof. Wei Bang Sun<br>Yunnan Innovation Team Program for conservation and utilization of PSESP (Plant Species with Extremely Small Populations) (2018HC005) — Prof. Wei Bang Sun<br>STS Program of the Chinese Academy of Sciences "Full Cover Conservation Project of Native Plants in Southwestern China" (KFJ-3W-No1) — Prof. Wei Bang Sun |
| **Abstract:** | Background: Acer yangbiense is a newly described critically endangered endemic maple tree confined to the Yangbi County in Yunnan Province in Southwest China. It was included in a programme for rescuing the most threatened species in China, focusing on 'plant species with extremely small populations'. Fingdings: We generated 64 Gb, 94 Gb and 110 Gb of raw DNA sequences and obtained chromosome-level genome assembly of A. yangbiense through a combination of Pacific Biosciences (PacBio) Single-molecule Real-time (SMRT), Illumina HiSeq X, and Hi-C mapping. The final genome assembly is approximately 666 Mb, with 13 chromosomes covering ~97% of the genome and scaffold N50 sizes of 45 Mb. Further, Benchmarking Universal Single-Copy Orthologs analysis recovered 95.5% complete BUSCO genes. The total number of repetitive elements account for 68.0% of the A. yangbiense genome. Genome annotation generated 30,418 protein-coding genes, assisted by a combination of prediction and transcriptome sequencing. In addition, nearly 1:1 orthology ratio of dot plots of longer syntenic blocks revealed a similar evolution history between A. yangbiense and grape, indicating that the genome has not undergone a whole genome duplication (WGD) event after the core-eudicot-common hexaploidization (ECH). Conclusion: Here, we report a high quality de novo genome assembly of A. yangbiense, the first genome for the genus Acer and the family Aceraceae. This will provide a fundamental conservation genomics resources as well as representing a new high-quality reference genome for the economically important Acer lineage and the wider order of Sapindales. |
| **Corresponding Author:** | Yongpeng Ma<br><br>CHINA |
| **Corresponding Author Secondary Information:** | |
| **Corresponding Author's Institution:** | |
| **Corresponding Author's Secondary** | |

Powered by Editorial Manager® and ProduXion Manager® from Aries Systems Corporation

| | |
|---|---|
| Institution: | |
| First Author: | Jing Yang |
| First Author Secondary Information: | |
| Order of Authors: | Jing Yang |
| | Hafiz Muhammad Wariss |
| | Li Dan Tao |
| | Ren Gang Zhang |
| | Quan Zheng Yun |
| | Peter Hollingsworth |
| | Zhi Ling Dao |
| | Gui Feng Luo |
| | Hui Jun Guo |
| | Yong Peng Ma |
| | Wei Bang Sun |
| Order of Authors Secondary Information: | |
| Additional Information: | |
| Question | Response |
| Are you submitting this manuscript to a special series or article collection? | No |
| **Experimental design and statistics**<br><br>Full details of the experimental design and statistical methods used should be given in the Methods section, as detailed in our Minimum Standards Reporting Checklist. Information essential to interpreting the data presented should be made available in the figure legends.<br><br>Have you included all the information requested in your manuscript? | Yes |
| **Resources**<br><br>A description of all resources used, including antibodies, cell lines, animals and software tools, with enough information to allow them to be uniquely identified, should be included in the Methods section. Authors are strongly encouraged to cite Research Resource Identifiers (RRIDs) for antibodies, model organisms and tools, where possible. | Yes |

| | |
|---|---|
| Have you included the information requested as detailed in our Minimum Standards Reporting Checklist? | |
| **Availability of data and materials**<br><br>All datasets and code on which the conclusions of the paper rely must be either included in your submission or deposited in publicly available repositories (where available and ethically appropriate), referencing such data using a unique identifier in the references and in the “Availability of Data and Materials” section of your manuscript.<br><br>Have you have met the above requirement as detailed in our Minimum Standards Reporting Checklist? | Yes |

Manuscript

Click here to download Manuscript Manuscript-Acer_yangbiense.docx

# *De novo* genome assembly of the endangered *Acer yangbiense*, a plant species with extremely small populations endemic to Yunnan of China

Jing Yang[1,2,†] , Hafiz Muhammad Wariss[1,2,3,†], Li Dan Tao[1,2], Ren Gang Zhang[4], Quan Zheng Yun[4], Peter Hollingsworth[5], Zhi Ling Dao[1,2], Gui Feng Luo[1,2,], Hui Jun Guo[6], Young Peng Ma[1,2*], Wei Bang Sun[1,2,7*]

[1]Yunnan Key Laboratory for Integrative Conservation of Plant Species with Extremely Small Populations, Kunming Institute of Botany, Chinese Academy of Sciences, Kunming, 650201, China, [2]Key Laboratory for Plant Diversity and Biogeography of East Asia, Kunming Institute of Botany, Chinese Academy of Sciences, Kunming, 650201, China, [3]University of Chinese Academy of Sciences, Beijing, 100049, China, [4]Beijing Ori-Gene Science and Technology Co. Ltd, Beijing, 102206, China, [5]Royal Botanic Garden Edinburgh, 20a Inverleith Row, Edinburgh, UK, [6]Southwest Forestry University, Kunming, 650224, Yunnan, China, [7]Kunming Botanical Garden, Kunming Institute of Botany, Chinese Academy of Sciences, Kunming, 650201, China,

*Correspondence should be addressed to Yong Peng Ma, E-mail: mayongpeng@mail.kib.ac.cn and Wei Bang Sun, E-mail: wbsun@mail.kib.ac.cn

[†]These authors contributed equally to this work.

**Abstract**

**Background:** *Acer yangbiense* is a newly described critically endangered endemic maple tree confined to the Yangbi County in Yunnan Province in Southwest China. It was included in a programme for rescuing the most threatened species in China, focusing on 'plant species with extremely small populations'. **Fingdings**: We generated 64 Gb, 94 Gb and 110 Gb of raw DNA sequences and obtained chromosome-level genome assembly of *A. yangbiense* through a combination of Pacific Biosciences (PacBio) Single-molecule Real-time (SMRT), Illumina HiSeq X, and Hi-C mapping. The final genome assembly is approximately 666 Mb, with 13 chromosomes covering ~97% of the genome and scaffold N50 sizes of 45 Mb. Further, Benchmarking Universal Single-Copy Orthologs analysis recovered 95.5% complete BUSCO genes. The total number of repetitive elements account for 68.0% of the *A. yangbiense* genome. Genome annotation generated 30,418 protein-coding genes, assisted by a combination of prediction and transcriptome sequencing. In addition, nearly 1:1 orthology ratio of dot plots of longer syntenic blocks revealed a similar evolution history between *A. yangbiense* and grape, indicating that the genome has not undergone a whole genome duplication (WGD) event after the core-eudicot-common hexaploidization (ECH). **Conclusion:** Here, we report a high quality de novo genome assembly of *A. yangbiense*, the first genome for the genus *Acer* and the family Aceraceae. This will provide a fundamental conservation genomics resources as well as representing a new high-quality reference genome for the economically important *Acer* lineage and the wider order of Sapindales.

***Keywords:*** *Acer yangbiense*; PSESP; PacBio sequencing; genome assembly; genome annotation

## Data description

### Background information

*Acer* L., commonly known as maple, is one of the most important genus of trees and shrubs in the Northern Hemisphere [1-5]. *Acer* exhibits a classical pattern of biogeographic disjunction across Europe, Northern Africa, Asia, and North America with the greatest species richness in Eastern Asia [2, 4, 6-12]. It is a wide-ranging genus comprising up to 129 species worldwide with maximum diversity in China, where about 99 (61 endemic, three introduced) species are recognized [13]. *Acer* has long been of interest to botanists for its remarkable diversity, especially of leaves, fruits, and bark, and for its intercontinental disjunct distribution [7]. The colorful foliage of maples is a charismatic landscape feature, with vivid hues of red, yellow, and orange in the autumn. In addition to being ornamental, many species are sources of commercial products, such as maple syrup, furniture and timber [14]. Maple has been found to contain a large number of phytochemicals which have antioxidant, antitumor and anti-inflammatory activities [14-19].

*Acer yangbiense* Y. S. Chen & Q. E. Yang (Aceraceae) is a newly described Chinese maple species (Fig. 1) [20]. It has a restricted distribution range between 2200-2500m altitudes in the western valley of Cangshan Mountain, Yunnan Province, China. This species is facing a very high

risk of extinction because of its small population size, poor reproduction, and habitat degradation [24]. The species was categorized as critically endangered (CR) by Gibbs and Chen in 2009 [21], and as only 5 individuals were recorded based on Qin et al. (2017) [22-25] in the first decade after its description. In 2016, further survey work recovered a total of 577 individuals from 12 localities [26]. This is the most accurate available population estimate of *A.yangbiensis*.

*A. yangbiensis*, is classified as a ‘plant species with extremely small populations’ (PSESP) by the Chinese government and included in the PSESP rescue plan [27, 28]. The concept of PSESP emphasizes species that face high risk of extinction, characterized by small remaining populations in restricted habitats and being subjected to severe human disturbance [27, 29]. It is targeted at species with less than 5000 mature individuals in total and fewer than 500 mature individuals in each isolated population [30]. Genetic studies done by Yang et al. (2015) suggested that *A. yangbiense* was not genetically depauperate, but further parentage analysis indicated a high selfing rate in seedlings of *A. yangbiense* [23]. The current threatened status of *A. yangbiense* serves to emphasize that an effective conservation strategy is urgently required.

The generation of plant genome sequences and assemblies allows detailed insights into the evolutionary history of species and provides information to support sustainable conservation [31]. Here, we presented a high-quality genome assembly of *A. yangbiense*, as a valuable resource and reference for future population genomic studies. The availability of a fully sequenced and annotated genome is essential to resolve fundamental questions about *A. yangbiense*

diversification and provide new insights into its demographic history, with important implications for future conservation efforts.

## Plant material

Fresh young leaves were collected from *ex-situ* conserved *A. yangbiense* at the Kunming Botanical Garden (KBG) of the Kunming Institute of Botany, Chinese Academy of Sciences. This tree was grown from seed in 2009, from seeds originally collected from Malutang, Yangbi County, Dali, Yunnan (**Fig. 1**) (N 25.7489 latitude, E 100.0064 longitude, 2474m elevation). For genome library preparation, only leaf tissues was used; for transcriptome sequencing, samples were obtained from 5 different tissues: leaf buds, young leaves, young stems, roots and fruits from healthy individuals planted in KBG in June and July 2018 respectively. All samples were collected with permission from KBG. For RNA samples, tissues were immediately transferred into liquid nitrogen and stored in dry ice until RNA extraction; for DNA samples, tissues were immediately stored in dry ice until DNA extraction.

## PacBio SMRT sequencing

Genomic DNA with high-quality and high-molecular-weight was extracted from fresh leaves using a CTAB protocol [32]. Libraries for single molecule real-time (SMRT) PacBio genome sequencing were constructed following the standard protocols of Pacific Biosciences at Beijing Ori-Gene

Science and Technology Co., Ltd (Beijing, China). Briefly, 50 μg of high quality genomic DNA was sheared to ~20 kb targeted size, followed by damage repair and end repair, blunt-end adaptor ligation, and size selection. Finally, the libraries were sequenced on the PacBio Sequel platforms using S/P2-C2 sequencing chemistry (10 SMRT cells). A total of 6.3 M PacBio reads with ~64 GB sequencing data were generated, with an average read length of 10Kb. The longest read was 93 Kb and N50 was 16.8 Kb (**Supplementary Table S1**).

## Illumina sequencing

The Illumina libraries were constructed according to the standard manufacturer's PCR-free protocol (Illumina). Short-insert libraries of 300-500-bp were prepared using 2 μg of whole genomic DNA for Illumina sequencing. All the libraries were sequenced on Illumina HiSeq Xplatform with pair-end sequencing strategy. In total, three PCR-free libraries were generated, and Fastp v0.19.3 [33] was used to filter out low quality reads and adaptor sequences. A total of 624.149 million raw reads was generated. This produced ~94.246 Gb (roughly 140x the assembled genome) of raw sequencing data, with an average cleaned read length of 148.5 bp (**Supplementary Table S2**).

## Hi-C sequencing

The Hi-C library was prepared by Beijing Ori-Gene Science and Technology Co., Ltd (Beijing, China) with the standard procedure described as follows. 700ng of high molecular-weight genomic DNA was cross-linked *in situ*, extracted, and then digested with a restriction enzyme. The sticky ends of the digested fragments were biotinylated, diluted, and then ligated to each other randomly. Biotinylated DNA fragments were enriched and sheared to a fragment size of 300-500 bp again for preparing the sequencing library, which was sequenced on a HiSeq X Ten platform (Illumina). A total of 740 M reads with ~110 GB sequencing data were generated (roughly 170x the assembled genome) with an average read length of 149.8 bp **(Supplementary Table S3).** During preprocessing of the Illumina data, Fastp v0.19.3 [33] was used to remove the short reads, low quality and adapter sequences.

**Estimation of genome size, heterozygosity, and repeat content**

Three short fragment libraries were constructed by PCR-free method and the Whole-Genome-Shotgun (WGS) short reads were generated using Illumina HiSeq X Ten machine, which were filtered and corrected with Fastp v0.19.3 [33].The genome size of *A. yangbiense* was estimated by the K-mer method [34] using sequencing data from the Illumina DNA library. Firstly, Jellyfish v2 (Jellyfish, **RRID: SCR_005491**) [34] was used to count the occurrence of k-mers based on the processed data. Finally, gce v1.0.0 [35] was used to estimate the overall characteristics of the genome, such as genome size, repeat contents, and level of heterozygosity. In this study,

67,781,536,308 k-mers were generated, and the peak k-mer depth was 111 (**Supplementary Fig.S1**). The genome size was estimated to be approximately 640 Mb, and repeat and heterozygosity rates were estimated to be 68.75% and 0.19% respectively based on k-mer individuals (**Supplementary TableS4)**.

### *De novo* assembly and chromosome construction

The *do novo* genome assembly was performed on full PacBio long reads using different assembly strategies to obtain a better genome assembly. Primary assembly v0.1 was generated from PacBio long reads by Canu v1.7 [36], assembly v0.2 by SMARTdenovo v1.0 [37], assembly v0.3 by Wtdbg v1.2.8 [38], assembly v0.4 after correction by Canu v1.7 [36] and SMARTdenovo v1.0 [37], assembly v0.5 after corrected by Canu v1.0 [36] and Wtdbg v1.2.8 [38], assembly v0.6 after corrected and trimmed by Canu v1.7 [36] + SMARTdenovo v1.0 [37], and assembly v0.7 after corrected and trimmed by Canu v1.7 + Wtdbg v1.2.8 [38] (**Supplementary Table S5**). The assembly (v0.4) from SMARTdenovo v1.0 [37] after Canu v1.7 [36] correction was chosen as the optimal assembly for further polishing and scaffolding. In this selected primary assembly (v0.4), the assembled genome size was 666 Mb distributed across 880 contigs with N50 of 2.3 Mb, L50 of 84, and maximum contig length of 11.9 Mb **(Supplementary Table S5)**. The draft assembly was first polished with Pilon v1.22 (Pilon, **PRID: SCR_014731**) [39] based on the high-quality Illumina sequencing reads and then piped into the Hi-C assembly workflow. Clean Hi-C reads

were mapped to the draft assembly with Juicer [40], and then a candidate chromosome-length assembly was generated automatically using the 3d-DNA pipeline to correct mis-joins, order, orient and anchor contigs from the draft assembly [41]. Manual review and refinement of the candidate assembly was performed in Juicebox Assembly Tools (JBAT) [42] for quality control and interactive correction. To reduce the influence of interactions of chromosomes and to further improve the chromosome-scale assembly, each chromosome was re-scaffolded with 3d-DNA [41] separately, and then manually refined with Juicebox [43]. With the modified 3d-DNA and JBAT workflow, 13 chromosomes (646206981 bp, ~97.04%) were anchored with only 265 contigs (18,721,930 bp) un-placed. Finally, after gap filling with LR_GapCloser v1.1 (GapCloser, **RRID: SCR_015026**) [44] (based on PacBio long reads, running for two rounds), Pilon v1.22 (Pilon, PRID: **SCR_014731**) was used to polish the assembly (based on Illumina reads, running for five rounds), and Redundans v0.13 [45] was used to slightly remove the redundancy of un-anchored sequences, to obtain the final genome assembly (v1.1) (**Supplementary Table S5**). In this final genome assembly v1.1 (**Supplementary Table S5**), we achieved an assembled genome size of 666 Mb characterized by 562 contigs and 280 scaffolds (with contig N50 of 5.5 Mb and scaffold N50 of 45 Mb) (**Table 1 and Supplementary Table S5**).

**Assessment of genome assembly**

We evaluated the level of genome completeness of the final genome assembly using Benchmarking Universal Single-Copy Orthologs (BUSCO, **RRID: SCR_015008**) [46] and the LTR Assembly Index (LAI) [47]. BUSCO analysis showed that 95.5% (90.8% complete and single-copy genes and 4.7% complete and duplicated genes) and 2.2% of the 1440 expected embryophytic genes were identified as complete and fragmented genes, respectively (**Table 2**). In addition, a relatively high LAI score = 12.21 (categorized as Reference level when: 10 ≤ LAI ≤20) showed that the assembly yielded high sequence continuity [47], agreeing with the BUSCO completeness. **(Supplementary table S5**). The overall map ratio of transcriptome data was 95.0% by HiSat2 v2.1.3 [48], showing good completeness of the assembly. The mapping of the whole Illumina short reads and bases by BWA v0.7.17-r1188- (BWA, **RRID: SCR_010910**) [49] were 99.4% for both, which means almost all sequencing data were represented (covering 98.4% of the total genome length, among which, 97.9% with a coverage depth ≥ 5×, 97.7% with a coverage depth ≥ 10×, 97.4% with a coverage depth ≥ 20×, showing high coverage). Meanwhile, mapping of PacBio reads and bases by minimap2 v2.11-r797 [50] were 76.5% and 94.3% respectively (covering 99.98% of the total length of genome, among which, 99.9% with a coverage depth ≥ 5×; 99.8% with a coverage depth≥ 10 ×; 99.4% with a coverage depth ≥ 20×). Both coverage rates of Illumina sequencing and PacBio sequencing were consistent and relatively high. The coverage depth distribution of the whole genome, as well as both gene regions of single-copy and duplicated BUSCO core genes was plotted. The duplicated genes had the same depth distribution as the

single-copy genes, indicating that the duplicated genes were not derived from unmerged haplotigs and thus there was almost no redundancy in the assembly (**Supplementary Fig. S2**). SAMtools (SAMtools, **RRID: SCR_005227**) [51] were used to detect variant sites. Heterozygosity rate were calculated by heterozygosity sites, and error rate of single bases was calculated by homozygosity sites. The heterozygosity rate was about 0.097% while the error rate was about 0.0037%. A GC depth analysis was conducted to assess potential contamination during sequencing and the coverage of the assembly, revealing that the genome had an average GC content of 35.96% with no obvious GC bias (**Supplementary Fig. S3**). We searched all sequences of the genome assembly against the NCBI non-redundant nucleotide database (NT) with BLASTN to assess contamination, and the results suggested no potential contamination. Mapping the Hi-C data to the final genome assembly using Juicer [5], the cluster results showed there were 13 unambiguous chromosome scaffolds with no obvious chromosome assembly error (**Supplementary Fig. S4).**

**DNA repeats annotation**

To de novo identify and classify repeat families in the genome assembly, the software package RepeatModeler v1.0.8 (RepeatModeler, **RRID: SCR_015027**) [52] was used with two complementary computational methods for de novo identifying repeats within the genome: RECON v1.08 and RepeatScout v1.0.5 (RepeatScout, **RRID: SCR_014653**). Then, using the output data file from RepeatModeler as a custom repeat library, RepeatMasker v4.0.7

(RepeatMasker, **RRID: SCR_012954**) [53] was employed to screen for repeats within the assembled genome. In summary, repeat sequences were estimated to account for 68.0% (452.81 Mb) of the *A. yangbiense* assembly, among which 17.32% were uncharacterized repeats. Long terminal repeats (LTRs) were dominant (250.98 Mb, 37.7%) with Copia (179.64 Mb) and Gypsy (66.18 Mb), the most abundant subtypes, representing 26.98% and 9.94% of the genome assembly, respectively. The results of repeat annotations are summarized in **Supplementary Table S6.**

## Transcriptome Assembly

Total RNA was extracted from the stem, roots, fruits, buds and leaves using the Trizol reagent according to manufacturer's instructions (Invitrogen). RNA quality was assessed on a Nanodrop-2000 spectrophotometer. The paired-end (PE) RNA-seq libraries were prepared using the NEBNEXT Ultra RNA Library Prep Kit for Illumina, and 150 bp PE sequencing was performed on an Illumina HiSeq X Ten platform. A total of 252.03 million raw reads were generated (**Supplementary Table S7**). Using HiSat2 v2.1.0 (HiSat2, **RRID: SCR_015530**) [48], raw reads from RNA sequencing were aligned to the genome assembly. Then reference genome-guided transcriptome assemblies were constructed with StringTie v1.3.5 (StringTie, **RRID: SCR_016323**) [54] and Trinity v2.0.6 (Trinity, **RRID: SCR_013048**) [55] respectively. De novo assembly was generated using Trinity. After that, transcriptome assemblies were combined and further refined with CD-HIT v4.6 (CD-HIT, **RRID: SCR_007105**) [56]. In the end, a 138.40 Mb transcriptome

with 82,766 unique transcripts were obtained as RNA-seq evidence in genome annotation. The summary is shown in **Supplementary Table S8**.

## Genome Annotation

The Maker2 genome annotation pipeline [57] was employed to predict protein-coding genes. After masking the repetitive sequences, AUGUSTUS v3.3.1 (AUGUSTUS, **RRID: SCR_008417**) [58] was used for *ab initio* gene prediction with model training based on 1,248 single copy orthologs, which were predicted by BUSCO (BUSCO, **RRID: SCR_015008**) [59] from the genome assembly. Then, for evidence-based gene prediction, transcripts from RNA sequencing were aligned to the repeat-masked reference genome assembly with BlastN (BLASTN, **RRID: SCR_001598**) and TblastX (TBLASTX, **RRID: SCR_011823**) from BLAST v2.2.28+ (NCBI BLAST, **RRID: SCR_004870**) [58]; protein sequences from *Arabidopsis thaliana* and *Dimocarpus longan* were aligned to the repeat-masked reference genome assembly with BlastX (BLASTX, **RRID: SCR_001653**). After optimization with Exonerate v2.2.0 (Exonerate, **RRID: SCR_016088**) [60], MAKER package v2.31.9 (MAKER, **RRID: SCR_005309**) [57], were used to prepare gene model predictions. AED (Annotation Edit Distance) scores were generated for each of the predicted genes as part of the MAKER pipeline, in order to assess the quality of gene prediction. Non-coding RNAs in the genome were identified by searching from the Rfam database [61]. tRNAscan-SE [62] and RNAMMER [63] were used to predict tRNAs and rRNAs,

respectively. Gene sets were integrated into a non-redundant gene annotation, and its completeness was checked using BUSCO (the 1,440 single-copy orthologs from the embryophyta_odb9 database) [46].

From the assembled genome of *A. yangbiense* a total of 30,418 genes were annotated. Besides, 28,320 protein-coding genes were acquired, with 25,572 of which had an AED<0.5 and a mean of 5.36 exons per gene. The average lengths of gene region, transcript and coding DNA sequence were 3,880 bp, 1,455 bp, and 1,308 bp, respectively (**Supplementary Table S9**). With regard to noncoding RNA, 734 ncRNA, 248 rRNA and 1116 tRNA were identified by Rfam, RNAMMER and tRNAScan-SE, respectively. The BUSCO evaluation showed that 95.5% of 1,440 expected plant genes were identified as complete (**Table 2**).

Gene function annotation was assigned based on sequence and domain conservation. For assignment based on sequence conservation, a BLAT (E-value shreshold of $1e^{-5}$) (BLAT, **RRID: SCR_011919**) [64] search of the peptide sequences from several protein databases was performed, such as Swiss-Prot [65, 66], TrEMBL [65, 67], NR [68], Pfam [69], and eggnog [70]. Assignment based on domain conservation: InterProScan (InterProScan, **RRID: SCR_005829**) [71] was used to examine motifs and domains by matching against public databases, such as ProDom [72], PRINTS [73], Pfam, SMART [74], PANTHER [75] and PROSITE [76]. As a result, 26,257 (92.7%) and 27,827 (98.26%) protein-coding genes out of the total 28,320 genes in the *A.*

*yangbiense* genome were assigned potential functions by BLAT and InterProScan, respectively (**Supplementary Table S10).**

**Identification of orthologous genes and phylogenetic tree construction**

OrthoMCL v2.0.9 (Ortholog Groups of Protein Sequences, **RRID: SCR_007839**) [77] was used to identify orthologous and paralogous gene clusters in the assembled genomes of *A. yangbiense* and 14 related plant species (**Supplementary Table S11**), including *Arabidopsis thaliana* [78], *Theobroma cacao* [79], *Citrus grandis* [80], *Populus trichocarpa* [81], *Eucalyptus grandis* [82], *Vitis vinifera* [83, 84], *Coffea canephora* [85], *Beta vulgaris* [86], *Carica papaya*[87], *Dimocarpus longan* [88], *Fragaria vesca* [89], *Medicago truncatula* [90], *Sclerocarya birrea* [91] and *Oryza sativa* [92]. Recommended settings were used for all-against-all BLASTP comparisons (Blast+v2.3.056) [58] and OrthoMCL [93] analysis.

A total of 29,892 OrthoMCL families including 379,261 genes were built based on effective database sizes of all vs all BLASTP with an E-value of $10^{-5}$ and a Markov chain clustering default inflation parameter. Additionally 542 gene families with 1,793 genes were identified to be specific to the *A. yangbiense* genome when compared with the other 14 genomes (**Supplementary Table S12**). Furthermore *A. yangbiense* and *D. longan* had the largest number of shared gene families (12,505) among the studied plants, supporting the closer relative relationships in the same family

of Sapindaceae compared with other plant species (phylogeny of the angiosperms, APG IV) ([94] accessed at 22 January 2019).

Phylogenetic analysis was performed using 854 orthologous protein coding single-copy genes among the 15 genomes found by OrthoMCL [93]. These were then aligned with MUSCLE v3.8.31 (MUSCLE, **RRID: SCR_011812**) [95]. A maximum likelihood phylogenetic tree was then generated using the concatenated amino acid sequences in PhyML v3.0 with the default parameter (LG Model). [96]. The divergence time was estimated with r8s v1.81 [97] and calibrated against the divergence timing of Monocotyledoneae and Eudicotyledoneae (synchronously 135-130 million years), of Pentapetalae (126-121 Ma), and Rosidae (123-115 Ma) [98]. The time calibrated tree was further analyzed together with these shared orthologous gene families among 15 plants by CAFE v4.0 [99], to detect expansion, contraction and rapid evolution of those observed gene families.

The phylogenetic analysis identified the closest relationship of *A. yangbiense* to *D. longan*, with the divergence time between them estimated at~31.11 Mya (**Fig. 2a**). Moreover the close relationship among Sapindaceae, Anacardiaceae (*S. birrea*) and Rutaceae (*C. grandis*) were confirmed, supporting the placement of the three families within the order of Sapindales in APG IV (**Fig. 2a**). Using CAFE v4.0 [99], a total of 1169 gene families were detected that have expanded, while 1,392 gene families were found to have contracted in *A. yangbiense*. The expanded gene families were enriched for 209 significant (q <0.05) GO terms of three functional

categories, i.e., BP (Biological Process), CC (Cellular Component), and MF (Molecular Function) (**Supplementary Table S13**), and five KEGG (Kyoto Encyclopedia of Genes and Genomes) pathways (**Supplementary Table S14**) significant at q <0.05. Alternatively the contracted gene families were enriched for 334 GO-terms of the mentioned-above three functional categories (**Supplementary Table S15**) and 14 KEGG pathways (**Supplementary Table S16**) involving several aspects of secondary metabolism, at q <0.05. Additionally, functional enrichment analysis of rapidly evolving gene families reveal 218 significant GO terms and 17 KEGG pathways, both at q <0.05, which were summarized in **Supplementary Table S17** (for GO enrichment) and **Supplementary Table S18** (for KEGG enrichment).

**Genome evolution by synteny analysis**

We performed synteny analysis of orthologous and paralogous genes previously identified by OrthoMCL [18] from *A. yangbiense* genomes, using MCScanX with default parameters, requiring at least five gene pairs per syntenic block [100]. The resulting dot plots were additionally employed to assess characteristics of syntenic blocks by comparison within and between genomes (grape).

The Ks value was calculated to determine possible events of WGD and/or other duplications like TE. First, protein sequences of those homologous colinear genes from *A. yangbiense* versus grape identified by MCScanX [100] were aligned against each other with MUSCLE (MUSCLE, **RRID: SCR_011812**) [95] to achieve the conserved protein sequences of each species, which

were then conversed into the corresponding codon alignments implemented in PAL2NAL [101]. Finally, Ks values were calculated by KaKs_Calculator [102] with YN model [103]. Based on the genome construction of the most recent ancestor of flowering plants, referred to as the ancestral eudicot karyotype (AEK) by Murat et al. (2017) [104], we compared the maple genome to AEK and then painted the syntenic AEK blocks onto *A. yangbiense* chromosomes.

A total of 999 colinear gene pairs on 139 colinear blocks were inferred within the *A. yangbiense* genome. There are 10,144 colinear gene pairs from 452 colinear blocks were detected between *A. yangbiense* and grape (**Supplementary Table S19**). Dot plots of longer syntenic blocks between *A. yangbiense* and grape revealed a nearly 1:1 orthology ratio, indicating a similar evolution history to grape without undergoing a WGD event after the core-eudicot-common hexaploidization (ECH) [105]. Synonymous substitution rate (Ks) distributions of syntenic blocks for *A. yangbiense* paralogs and orthologs with other eudicots also support the hypothesis of no recent WGD event (**Fig. 2b, c, d**). However other than WGD, TE duplications might occur as the existence of short syntenic blocks in *A. yangbiense* (**Fig. 2b**). Furthermore the genome painter image by painting the syntenic AEK blocks onto *A. yangbiense* chromosomes illustrate that chromosomes 4,6,8 and 9 nearly exclusively contain the ancestral eudicot chromosome 2, 6, 5 without existence of inter-chromosomal segments (**Fig. 2e**). Such conserved gene content and order on these chromosomes in *A. yangbiense* could be due to the merged ancestral chromosome structures (e.g. multiple telomeres and centromeres on one chromosome) suppressing

recombination and/or successive rearrangement, as was simultaneously inferred from the genome of *E. grandis* [82]. Lastly we recommend that the genome of *A. yangbiense* has the potential to replace grape as the reference genome for studying recent WGD and chromosome evolution especially for species within and/or close relatives to the order of Sapindales, due to high quality of genome assembly, no recent WGD, as well as lower recombination of chromosomes in *A. yangbiense*.

## Conclusion

We have presented a *de novo* genome assembly of *A. yangbiense* using a combination of PacBio (SMRT), Illumina HiSeq X, and Hi-C approaches, and achieved a high-quality sequence assembly. The *A. yangbiense* genome that we have sequenced, assembled, and annotated here is the first genome for the genus *Acer* and the family Aceraceae. This critically threatened species genome will facilitate the genome assembly and resequencing of additional species. It will be an essential resource for further investigations of the demography, adaptability and conservation genetics of this endangered species. Likewise, the novel genome data generated in the present study will provide a valuable resource for studying the WGD and chromosome evolution particularly in the Sapindales.

## Availability of supporting data

The genome assembly, annotations, and other supporting data are available via the GigaScience database GigaDB. The raw sequence data have been deposited in the Short Read Archive under NCBI BioProject ID PRJNA524417.

## Additional files

**Figure S1.** Frequency distribution of the 17-mer graph analysis used to estimate the size of the *A. yangbiense* genome.

**Figure S2.** Length distribution of PacBio subreads. Assessment of the distribution of genome reads (Left) and BUSCO core region (right) coverage depth through PacBio-SMRT (lower) and Illumina sequencing data (upper).

**Figure S3.** Coverage depth of PacBio and Illumina sequencing data under different GCs. Assessment of the distribution of GC content and sequencing depth by PacBio-SMRT (left) and Illumina (right) under different GCs.

**Figure S4.** Hi-C map of final assembly of chromosomes. The distribution of links among chromosomes was exhibited by heatmap based on HiCplotter. The color key of heatmap ranging from light yellow to dark red indicated the frequency of Hi-C interaction links from low to high (0~10).

**Table S1.** WGS-PacBio sequencing statistics.

**Table S2.** WGS Illumina sequencing statistics.

**Table S3.** Hi C sequencing statistics.

**Table S4.** K mer survey statistics.

**Table S5.** Statistics of all assemblies.

**Table S6.** Repeat annotations of the *Acer yangbiense* genome assembly.

**Table S7.** Summary of Illumina RNA sequencing data.

**Table S8.** Summary of the transcriptome assemblies.

**Table S9.** Gene annotation statistics of the of *A. yangbiense* assembly.

**Table S10.** Functional annotation of predicted genes in *A. yangbiense* genome.

**Table S11.** Basic information with regards to genomes of 15 plants that were employed to gene family analysis and the phylogenetic tree construction.

**Table S12.** Summary of the gene family analyses. Unique groups and genes, single-copy and duplicated groups and genes are summarized for the 15 plant genomes.

**Table S13.** GO enrichment of expanded gene families. (A) ‘Category’ is the Gene Ontology (GO) term ID; (B) ‘P value’ is the over represented P-value indicating the observed frequency of a given term among analyzed genes is equal to the expected frequency based on the null distribution; i.e., lower P-values indicate stronger evidence for overrepresentation; (C) ‘Q value’ is the Benjamini and Hochberg adjusted P-value, (D) ‘numEPInCat’ is the number of expanded gene families in the corresponding GO category; (E) ‘numInCat’ is the number of detected gene families in the corresponding GO category; (F) ‘Term’ is the GO term; (G)‘Ontology’ indicates which ontology the term comes from. Significant biological significance is at $q < 0.05$.

**Table S14.** KEGG enrichment of expanded gene families. (A) ‘KO category’ is the KEGG Orthology (KO) category ID; (B) ‘P value & Q value’ have the same meaning stated as Supplemental Table S13 (B) & (C); (D) ‘numEPInCat’ is the number of expanded gene families in the corresponding KO category; (E) ‘numInCat’ is the number of detected gene families in the corresponding KO category; (F) ‘Pathway’ is the KEGG pathway; (G) ‘Class’ indicates which KEGG class the pathway comes from. Significant biological significance is at q < 0.05.

**Table S15.** GO enrichment of contracted gene families.

**Table S16.** KEGG enrichment of contracted gene families.

**Table S17.** GO enrichment of rapidly evolved gene families.

**Table S18.** KEGG enrichment of rapidly evolved gene families.

**Table S19.** Summary of colinear analysis within and between species.

## Abbreviations

AED: Annotation Edit Distance; AEK: Ancestral Eudicot Karyotype; Blast: Basic Local Alignment Search Tool; BUSCO: Benchmarking Universal Single-Copy Orthologs; ECH: core-eudicot-common hexaploidization; GO: Gene Ontology; KEGG: Kyoto Encyclopedia of Genes and Genomes; LTR: long terminal repeat; Mya: million years ago; NCBI: National Center for Biotechnology Information; PSESP: plant species with extremely small populations; SMRT: Single Molecule Real-Time; TE: transposable element; WGD: Whole Genome Duplication

## Ethics approval and consent to participate

Not applicable. The collection of plant materials of this research was compliance with relevant local guidelines and appropriate permissions from Kunming Botanical Garden.

## Consent for publication

Not applicable.

## Competing interests

The authors declare that they have no competing interests.

## Funding

This study was funded by Science and Technology Basic Resources Investigation Program of China (Grant No. 2017FY100100), NSFC (National Natural Science Foundation of China)-Yunnan Joint Fund (Grant No. U1302262), National Key R&D Program of China (Grant No. 2017YFC0505200), Key Laboratory Construction of Yunnan Science and Technology Talents and Platform Program (Grant No. 2018DG004), Yunnan Innovation Team Program for conservation and utilization of PSESP (Plant Species with Extremely Small Populations) (Grant No. 2018HC005) and STS Program of the Chinese Academy of Sciences "Full Cover Conservation Project of Native Plants in Southwestern China" (KFJ-3W-No1).

## Author's contribution

W.B.S. and Y.P.M. designed the study; L.D.T. and G.F.L. collected and prepared the materials; R.G.Z and Q.Z.Y conducted the experiments and data analyzing. J.Y., H.M.W. and Y.P.M. wrote the

manuscript; H.P., L.D.T., Z.L.D., H.J.G. and W.B.S. revised the manuscript. All authors read and approved the final draft.

## Acknowledgement

The authors thank Dr. Alex Twyford from the University of Edinburgh for his help and comments from technical perspective.

## Competing interests

The authors declare that they have no competing interests.

# References


1. Ogata K. A systematic study of the genus *Acer*. Bulletin Tokyo University Forests; 1967.
2. de Jong PC. Flowering and sex expression in *Acer* L. A biosystematic study. Veenman; 1976.
3. Wu ZY, Raven PH, Hong DY. Flora of China. Vol. 11: Oxalidaceae through Aceraceae. Science Press, Beijing, and Missouri Botanical Garden Press, St. Louis; 2008.
4. van Gelderen DM, De Jong PC, Oterdoom HJ. Maples of the world. Timber Press; 1994.
5. Weakley A. Flora of the southern and mid-Atlantic states. University of North Carolina Herbarium; 2010.
6. Harris A, Chen Y, Olsen RT, et al. On merging *Acer* sections *Rubra* and *Hyptiocarpa*: Molecular and morphological evidence. PhytoKeys 2017;**86**:9-42.
7. Harris A, Frawley E, Wen J. The utility of single-copy nuclear genes for phylogenetic resolution of *Acer* and *Dipteronia* (Acereae, Sapindaceae). Annales Botanici Fennici 2017;**54**(4–6):209-22.
8. Wen J. Evolution of eastern Asian and eastern North American disjunct distributions in flowering plants. Annual Review of Ecology and Systematics 1999;**30**(1):421-55.
9. Wolfe JA, Tanai T. Systematics, phylogeny, and distribution of *Acer* (maples) in the Cenozoic of western North America. Journal of the Faculty of Science, Hokkaido University Series 4, Geology and Mineralogy 1987;**22**(1):1-246.
10. Renner SS, Beenken L, Grimm GW, et al. The evolution of dioecy, heterodichogamy, and labile sex expression in *Acer*. Evolution 2007;**61**(11):2701-19.

11. Renner SS, Grimm GW, Schneeweiss GM, et al. Rooting and dating maples (*Acer*) with an uncorrelated-rates molecular clock: implications for North American/Asian disjunctions. Systematic Biology 2008;**57**(5):795-808.

12. Huang SF, Ricklefs RE, Raven PH. Phylogeny and historical biogeography of *Acer* I-Study history of the infrageneric classification. Taiwania 2002;**47**(3):203-18.

13. Xu TZ, Chen YS, Piet CDJ, et al. Flora of China. Vol. 11: Aceraceae. Science Press, Beijing, and Missouri Botanical Garden Press, St. Louis; 2008.

14. Bi W, Gao Y, Shen J, et al. Traditional uses, phytochemistry, and pharmacology of the genus *Acer* (maple): A review. Journal of Ethnopharmacology 2016;**189**:31-60.

15. Ball DW. The chemical composition of maple syrup. Journal of Chemical Education 2007;**84**(10):1647-50.

16. Gonzalez-Sarrias A, Li L, Seeram NP. Anticancer effects of maple syrup phenolics and extracts on proliferation, apoptosis, and cell cycle arrest of human colon cells. Journal of Functional Foods 2012;**4**(1):185-96.

17. Perkins TD, van den Berg AK. Maple syrup-production, composition, chemistry, and sensory characteristics. Advances in Food and Nutrition Research 2009;**56**:101-43.

18. Legault J, Girard-Lalancette K, Grenon C, et al. Antioxidant activity, inhibition of nitric oxide overproduction, and in vitro antiproliferative effect of maple sap and syrup from *Acer saccharum*. Journal of Medicinal Food 2010;**13**(2):460-68.

19. Park KH, Yoon KH, Yin J, et al. Antioxidative and anti-inflammatory activities of galloyl derivatives and antidiabetic activities of *Acer ginnala*. Evidence-Based Complementary and Alternative Medicine 2017:1-8.

20. Chen YS, Yang QE, Zhu GH. *Acer yangbiense* (Aceraceae), a new species from Yunnan, China. Novon 2003;**13**(3):296-99.

21. Gibbs D, Chen YS. The red list of maples. Botanic Gardens Conservation International; 2009.

22. Qin HN, Yang Y, Dong SY, et al. Threatened species list of China's higher plants. Biodiversity Science 2017;**25**(7):696-744.

23. Yang J, Zhao LL, Yang JB, et al. Genetic diversity and conservation evaluation of a critically endangered endemic maple, *Acer yangbiense*, analyzed using microsatellite markers. Biochemical Systematics and Ecology 2015;**60**:193-98.

24. Zhao LL. *Genetic diversity of the critically endangered Yanbi maple*, *Acer yangbiense* (Aceraceae). Master dissertation, The Graduate School of Chinese Academy of Sciences, Beijing, 2011.

25. Zhao LL, Sun WB, Yang JB. Development and characterization of microsatellite markers in the critically endangered species *Acer yangbiense* (Aceraceae). American Journal of Botany 2011;**98**(9):e247-e49.

26. Tao LD. *Population ecology studies of two PSESP plants, and the reproductive biology and SSR primers of Acer yangbiense.* Master dissertation, The University of Chinese Academy of Sciences, Beijing, 2018.

27. Ma YP, Chen G, Grumbine RE, et al. Conserving plant species with extremely small populations (PSESP) in China. Biodiversity and Conservation 2013;**22**(3):803-09.

28. Sun WB, Yin Q. Conservation of the Yangbi maple *Acer yangbiense* in China. 2009.

29. Sun WB, Ma YP, Blackmore S. How a new conservation action concept has accelerated plant conservation in China. Trends in Plant Science 2019;**24**(1):4-6.

30. Sun WB. Words from the Guest Editor-in-Chief. Plant Diversity 2016;**38**(5):207-08.

31. Silva-Junior OB, Grattapaglia D, Novaes E, et al. Genome assembly of the Pink Ipê (*Handroanthus impetiginosus*, Bignoniaceae), a highly valued, ecologically keystone Neotropical timber forest tree. GigaScience 2018;**7**(1):gix125. doi:10.1093/gigascience/gix125.

32. Doyle JJ. A rapid DNA isolation procedure for small quantities of fresh leaf tissue. Phytochemical Bulletin 1987;**19**:11-15.

33. Chen S, Zhou Y, Chen Y, et al. fastp: an ultra-fast all-in-one FASTQ preprocessor. Bioinformatics 2018;**34**(17):i884-i90.

34. Marcais G, Kingsford C. A fast, lock-free approach for efficient parallel counting of occurrences of k-mers. Bioinformatics 2011;**27**(6):764-70.

35. Liu B, Shi Y, Yuan J, et al. Estimation of genomic characteristics by analyzing k-mer frequency in de novo genome projects. arXiv preprint arXiv 2013:1308-2012. doi:https://arxiv.org/abs/1308.2012.

36. Koren S, Walenz BP, Berlin K, et al. Canu: scalable and accurate long-read assembly via adaptive k-mer weighting and repeat separation. Genome Research 2017;**27**(5):722-36.

37. Ultra-fast de novo assembler using long noisy reads. https://github.com/ruanjue/smartdenovo. Accessed 01 October 2018.

38. A fuzzy bruijn graph (FBG) approach to long noisy reads assembly. https://github.com/ruanjue/wtdbg-1.2.8. Accessed 01 October 2018.

39. Walker BJ, Abeel T, Shea T, et al. Pilon: an integrated tool for comprehensive microbial variant detection and genome assembly improvement. PloS One 2014;**9**(11):e112963.

40. Durand NC, Shamim MS, Machol I, et al. Juicer provides a one-click system for analyzing loop-resolution Hi-C experiments. Cell Systems 2016;**3**(1):95-98.

41. Dudchenko O, Batra SS, Omer AD, et al. De novo assembly of the *Aedes aegypti* genome using Hi-C yields chromosome-length scaffolds. Science 2017;**356**(6333):92-95.

42. Dudchenko O, Shamim MS, Batra S, et al. The Juicebox Assembly Tools module facilitates de novo assembly of mammalian genomes with chromosome-length scaffolds for under $1000. bioRxiv preprint bioRxiv 2018:254797. doi:https://doi.org/10.1101/254797.

43. Durand NC, Robinson JT, Shamim MS, et al. Juicebox provides a visualization system for Hi-C contact maps with unlimited zoom. Cell Systems 2016;**3**(1):99-101.

44. Xu GC, Xu TJ, Zhu R, et al. LR_Gapcloser: a tiling path-based gap closer that uses long reads to complete genome assembly. GigaScience 2018;**8**(1):giy157. doi:https://doi.org/10.1093/gigascience/giy157.

45. Pryszcz LP, Gabaldon T. Redundans: An assembly pipeline for highly heterozygous genomes. Nucleic Acids Research 2016;**44**(12):e113.

46. Simao FA, Waterhouse RM, Ioannidis P, et al. BUSCO: assessing genome assembly and annotation completeness with single-copy orthologs. Bioinformatics 2015;**31**(19):3210-12.

47. Ou S, Chen J, Jiang N. Assessing genome assembly quality using the LTR Assembly Index (LAI). Nucleic Acids Research 2018;**46**(21):e126-e26.

48. Kim D, Langmead B, Salzberg SL. HISAT: a fast spliced aligner with low memory requirements. Nature Methods 2015;**12**(4):357-60.

49. Li H. Aligning sequence reads, clone sequences and assembly contigs with BWA-MEM. arXiv preprint arXiv 2013:1303-3997. doi:https://arxiv.org/abs/1303.3997.

50. Li H. Minimap2: fast pairwise alignment for long DNA sequences. arXiv preprint arXiv 2017:1708-01492. doi:https://arxiv.org/abs/1708.01492.

51. Li H, Handsaker B, Wysoker A, et al. The sequence alignment/map format and SAMtools. Bioinformatics 2009;**25**(16):2078-79.

52. Smit A, Hubley R: RepeatModeler Open-1.0. http://www.repeatmasker.org/RepeatModeler/. Accessed 01 October 2018.

53. Smit A, Hubley R, Green P: RepeatMasker Open-4.0 (2013-2015). http://repeatmasker.org. Accessed 01 October 2018.

54. Pertea M, Pertea GM, Antonescu CM, et al. StringTie enables improved reconstruction of a transcriptome from RNA-seq reads. Nature Biotechnology 2015;**33**(3):290-95.

55. Grabherr MG, Haas BJ, Yassour M, et al. Full-length transcriptome assembly from RNA-Seq data without a reference genome. Nature Biotechnology 2011;**29**(7):644-52.

56. Fu L, Niu B, Zhu Z, et al. CD-HIT: accelerated for clustering the next-generation sequencing data. Bioinformatics 2012;**28**(23):3150-52.

57. Cantarel BL, Korf I, Robb SM, et al. MAKER: an easy-to-use annotation pipeline designed for emerging model organism genomes. Genome Research 2008;**18**(1):188-96.

58. Boratyn GM, Schaffer AA, Agarwala R, et al. Domain enhanced lookup time accelerated BLAST. Biology Direct 2012;**7**(1):12.

59. Stanke M, Diekhans M, Baertsch R, et al. Using native and syntenically mapped cDNA alignments to improve de novo gene finding. Bioinformatics 2008;**24**(5):637-44.

60. Slater GSC, Birney E. Automated generation of heuristics for biological sequence comparison. BMC Bioinformatics 2005;**6**(1):31.

61. Griffiths-Jones S, Bateman A, Marshall M, et al. Rfam: an RNA family database. Nucleic Acids Research 2003;**31**(1):439-41.

62. Lowe TM, Eddy SR. tRNAscan-SE: a program for improved detection of transfer RNA genes in genomic sequence. Nucleic Acids Research 1997;**25**(5):955-64.

63. Lagesen K, Hallin P, Rodland EA, et al. RNAmmer: consistent and rapid annotation of ribosomal RNA genes. Nucleic Acids Research 2007;**35**(9):3100-08.

64. Kent WJ. BLAT-the BLAST-like alignment tool. Genome Research 2002;**12**(4):656-64.

65. Bairoch A, Apweiler R. The SWISS-PROT protein sequence database and its supplement TrEMBL in 2000. Nucleic Acids Research 2000;**28**(1):45-48.

66. ExPASy Bioinformatics Resources Portal. http://www.expasy.ch/sprot. Accessed 01 Dec 2017.

67. UniProt. http://www.ebi.ac.uk/uniprot. Accessed 01 Dec 2017.

68. National Center for Biotechnology Information. http://www.ncbi.nlm.nih.gov. Accessed 01 October 2018.

69. Punta M, Coggill PC, Eberhardt RY, et al. The Pfam protein families database. Nucleic Acids Research 2011;**40**(D1):D290-D301.

70. Jensen LJ, Julien P, Kuhn M, et al. eggNOG: automated construction and annotation of orthologous groups of genes. Nucleic Acids Research 2007;**36**(suppl_1):D250-D54.

71. Jones P, Binns D, Chang HY, et al. InterProScan 5: genome-scale protein function classification. Bioinformatics 2014;**30**(9):1236-40.

72. Corpet F, Gouzy J, Kahn D. Recent improvements of the ProDom database of protein domain families. Nucleic Acids Research 1999;**27**(1):263-67.

73. Attwood TK, Croning MD, Flower DR, et al. PRINTS-S: the database formerly known as PRINTS. Nucleic Acids Research 2000;**28**(1):225-27.

74. Schultz J, Copley RR, Doerks T, et al. SMART: a web-based tool for the study of genetically mobile domains. Nucleic Acids Research 2000;**28**(1):231-34.

75. Mi H, Lazareva-Ulitsky B, Loo R, et al. The PANTHER database of protein families, subfamilies, functions and pathways. Nucleic Acids Research 2005;**33**(suppl_1):D284-D88.

76. Sigrist CJ, De Castro E, Cerutti L, et al. New and continuing developments at PROSITE. Nucleic Acids Research 2012;**41**(D1):D344-D47.

77. Li L, Stoeckert CJ, Roos DS. OrthoMCL: identification of ortholog groups for eukaryotic genomes. Genome Research 2003;**13**(9):2178-89.

78. Cheng CY, Krishnakumar V, Chan AP, et al. Araport11: a complete reannotation of the *Arabidopsis thaliana* reference genome. The Plant Journal 2017;**89**(4):789-804.

79. Motamayor JC, Mockaitis K, Schmutz J, et al. The genome sequence of the most widely cultivated cacao type and its use to identify candidate genes regulating pod color. Genome Biology 2013;**14**(6):r53.

80. Wang X, Xu Y, Zhang S, et al. Genomic analyses of primitive, wild and cultivated citrus provide insights into asexual reproduction. Nature Genetics 2017;**49**(5):765-72.

81. Tuskan GA, Difazio S, Jansson S, et al. The genome of black cottonwood, *Populus trichocarpa* (Torr. & Gray). Science 2006;**313**(5793):1596-604.

82. Myburg AA, Grattapaglia D, Tuskan GA, et al. The genome of *Eucalyptus grandis*. Nature 2014;**510**(7505):356-62.

83. Jaillon O, Aury JM, Noel B, et al. The grapevine genome sequence suggests ancestral hexaploidization in major angiosperm phyla. Nature 2007;**449**(7161):463-67.

84. Canaguier A, Grimplet J, Di Gaspero G, et al. A new version of the grapevine reference genome assembly (12X. v2) and of its annotation (VCost. v3). Genomics Data 2017;**14**:56-62.

85. Denoeud F, Carretero-Paulet L, Dereeper A, et al. The coffee genome provides insight into the convergent evolution of caffeine biosynthesis. Science 2014;**345**(6201):1181-84.

86. Dohm JC, Minoche AE, Holtgrawe D, et al. The genome of the recently domesticated crop plant sugar beet (*Beta vulgaris*). Nature 2014;**505**(7484):546-49.

87. Ming R, Hou S, Feng Y, et al. The draft genome of the transgenic tropical fruit tree papaya (*Carica papaya* Linnaeus). Nature 2008;**452**:991-96.

88. Lin Y, Min J, Lai R, et al. Genome-wide sequencing of longan (*Dimocarpus longan* Lour.) provides insights into molecular basis of its polyphenol-rich characteristics. GigaScience 2017;**6**(5):1-14.

89. Shulaev V, Sargent DJ, Crowhurst RN, et al. The genome of woodland strawberry (*Fragaria vesca*). Nature Genetics 2011;**43**(2):109.

90. Young ND, Debelle F, Oldroyd GE, et al. The Medicago genome provides insight into the evolution of rhizobial symbioses. Nature 2011;**480**(7378):520-24.

91. Chang Y, Liu H, Liu M, et al. The draft genomes of five agriculturally important African orphan crops. GigaScience 2019;**8**(3):giy152. doi:https://doi.org/10.1093/gigascience/giy152.

92. Ouyang S, Zhu W, Hamilton J, et al. The TIGR rice genome annotation resource: improvements and new features. Nucleic Acids Research 2006;**35**(suppl_1):D883-D87.

93. Boetzer M, Henkel CV, Jansen HJ, et al. Scaffolding pre-assembled contigs using SSPACE. Bioinformatics 2010;**27**(4):578-79.

94. Angiosperm Phylogeny Website. http://www.mobot.org/MOBOT/Research/APweb/welcome.html. Accessed at 22 Jan 2019.

95. Edgar RC. MUSCLE: multiple sequence alignment with high accuracy and high throughput. Nucleic Acids Research 2004;**32**(5):1792-97.

96. Guindon S, Dufayard JF, Lefort V, et al. New algorithms and methods to estimate maximum-likelihood phylogenies: assessing the performance of PhyML 3.0. Systematic Biology 2010;**59**(3):307-21.

97. Sanderson MJ. r8s: inferring absolute rates of molecular evolution and divergence times in the absence of a molecular clock. Bioinformatics 2003;**19**(2):301-02.

98. Magallon S, Gomez Acevedo S, Sanchez Reyes LL, et al. A metacalibrated time- tree documents the early rise of flowering plant phylogenetic diversity. New Phytologist 2015;**207**(2):437-53.

99. De Bie T, Cristianini N, Demuth JP, et al. CAFE: a computational tool for the study of gene family evolution. Bioinformatics 2006;**22**(10):1269-71.

100. Wang Y, Tang H, DeBarry JD, et al. MCScanX: a toolkit for detection and evolutionary analysis of gene synteny and collinearity. Nucleic Acids Research 2012;**40**(7):e49-e49.

101. Suyama M, Torrents D, Bork P. PAL2NAL: robust conversion of protein sequence alignments into the corresponding codon alignments. Nucleic Acids Research 2006;**34**(suppl_2):W609-W12.

102. Zhang Z, Li J, Zhao XQ, et al. KaKs_Calculator: calculating Ka and Ks through model selection and model averaging. Genomics, Proteomics & Bioinformatics 2006;**4**(4):259-63.

103. Yang Z, Nielsen R. Estimating synonymous and nonsynonymous substitution rates under realistic evolutionary models. Molecular Biology and Evolution 2000;**17**(1):32-43.

104. Murat F, Armero A, Pont C, et al. Reconstructing the genome of the most recent common ancestor of flowering plants. Nature Genetics 2017;**49**(4):490-96.

105. Paterson AH, Wendel JF, Gundlach H, et al. Repeated polyploidization of Gossypium genomes and the evolution of spinnable cotton fibres. Nature 2012;**492**(7429):423-27.

## Tables

**Table 1:** *A. yangbiense* final genome assembly statistics

| | Contig | | Scaffold | |
|---|---|---|---|---|
| | Size(bp) | Number | Size(bp) | Number |
| **Total size** | 665887899 bp | 562 | - | 280 |
| **Total number** | **-** | - | - | - |
| **N10** | 10447168 bp | 5 | 73781861 bp | 1 |
| **N50** | 5479097 bp | 39 | 44917698 bp | 6 |
| **N90** | 835514 bp | 154 | 36383401 bp | 12 |
| **Max.** | 17438070 bp | - | 73781861 bp | - |
| **Min.** | 7640 bp | - | 7640 bp | - |
| **Mean** | 1184817 bp | - | 2378171 bp | - |
| **Median** | 137049 bp | - | 50985 bp | - |
| **Gap** | **-** | - | - | 282 |
| **GC content%** | 35.96% | - | - | - |

**Table 2:** Summary of BUSCO evaluation of the gene prediction

| | BUSCO groups | Percentage (%) |
|---|---|---|
| **Complete BUSCOs** | 1,375 | 95.5 |
| **Complete and single-copy BUSCOs** | 1,308 | 90.8 |
| **Complete and duplicated BUSCOs** | 67 | 4.7 |
| **Fragmented BUSCOs** | 29 | 2.0 |
| **Missing BUSCOs** | 36 | 2.5 |
| **Total BUSCO groups searched** | 1,440 | 100.00 |

## Figures

**Figure 1:** Images of *Acer yangbiense* chromosmes assembly, distribution range, flowers, fruits, and ex-situ conserved tree. **(a)** High-quality assembly of thirteen chromosomes. The tracks represent assembled 13 chromosomes, track depict Class I TE (LTR, LINE and SINE) density, Class II TE (DNA and Heliron) density, genes (mRNA) density, heterozygous (SNP and InDel) density, GC content and the curves of the innermost circle represent genome rearrangement events of collinear blocks. **(b)** Red-shaded regions denote distribution range of *A. yangbiense* in Yangbi county. **(c)** staminate inflorescence. **(d)** pistillate inflorescence. **(e)** fruits. **(f)** ex-situ conserved tree.

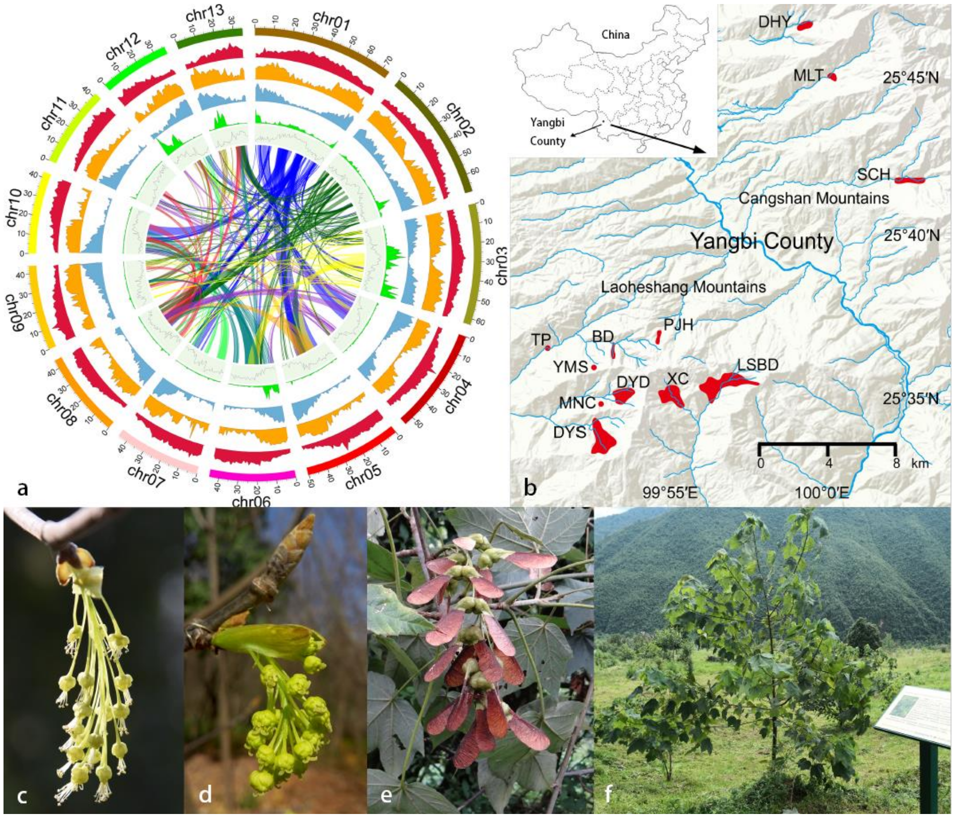

**Figure 2:** Genome evolution analysis of *A. yangbiense*. **(a)** Phylogenetic tree, divergence time, and profiles of gene families that underwent expansion or contraction; Comparison of dot plots of syntenic blocks within *A. yangbiense*. **(b)** and between *A. yangbiense* and grape genomes **(c)**. **(d)** Synonymous substitution rate (Ks) distributions of syntenic blocks for *A. yangbiense* paralogs and orthologs with other eudicots are represented. **(e)** Comparison with ancestral eudicot karyotype (AEK) chromosomes reveals synteny. The syntenic AEK blocks are painted onto *A. yangbiense* chromosomes.

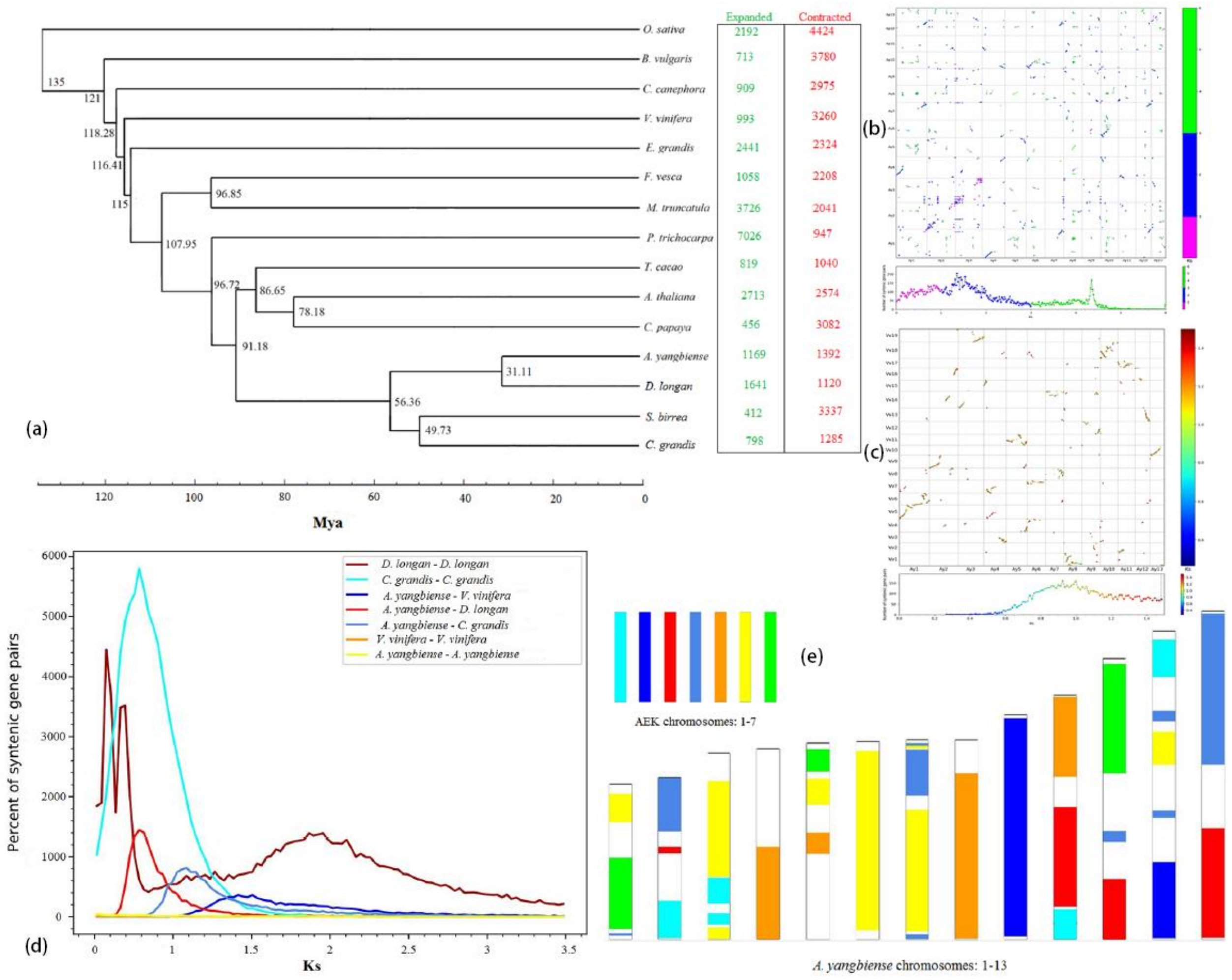

Figure 1 in MS
Click here to download Figure Fig_1.pdf

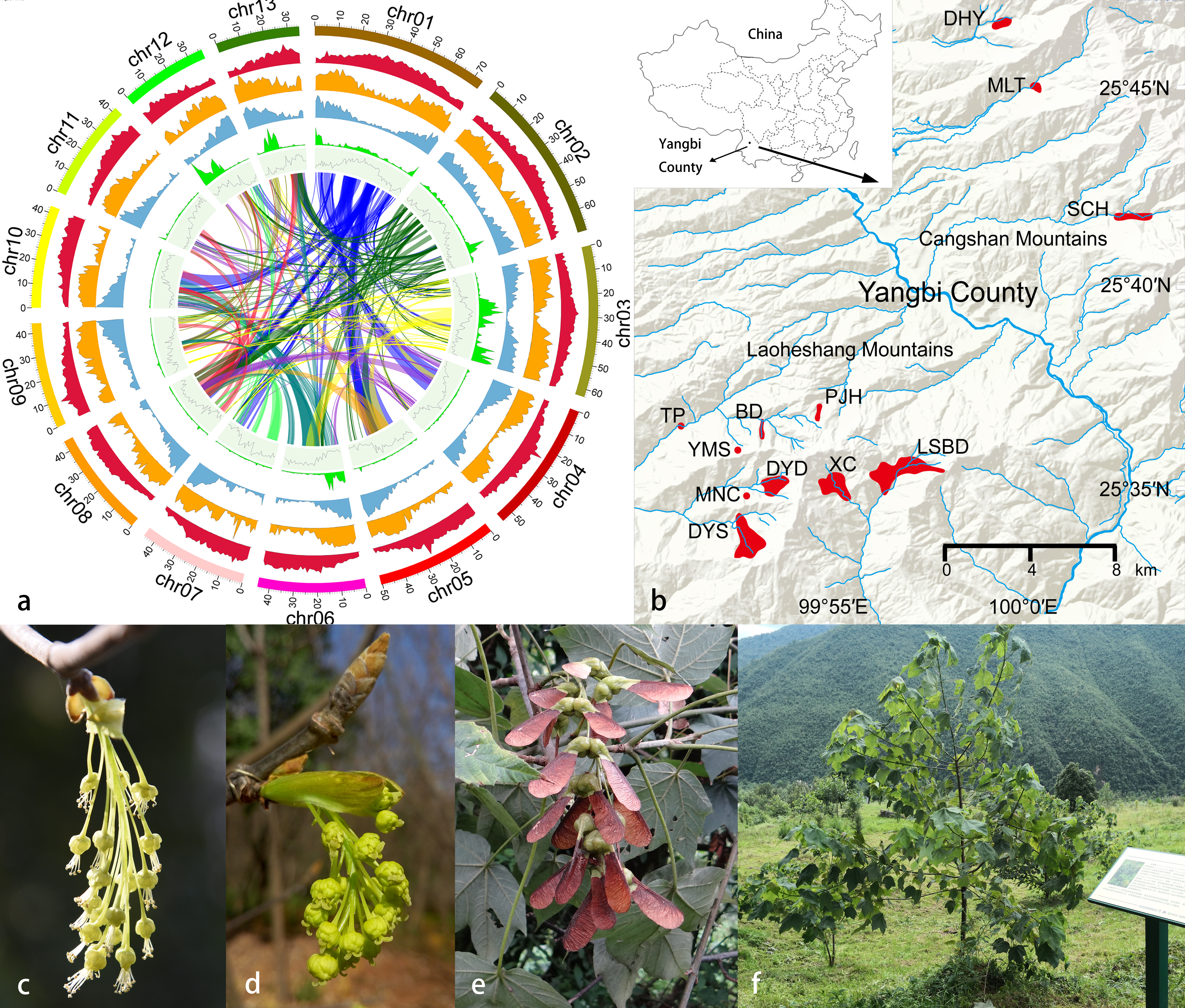

Figure 2 in MS

Click here to download Figure Fig_2.pdf

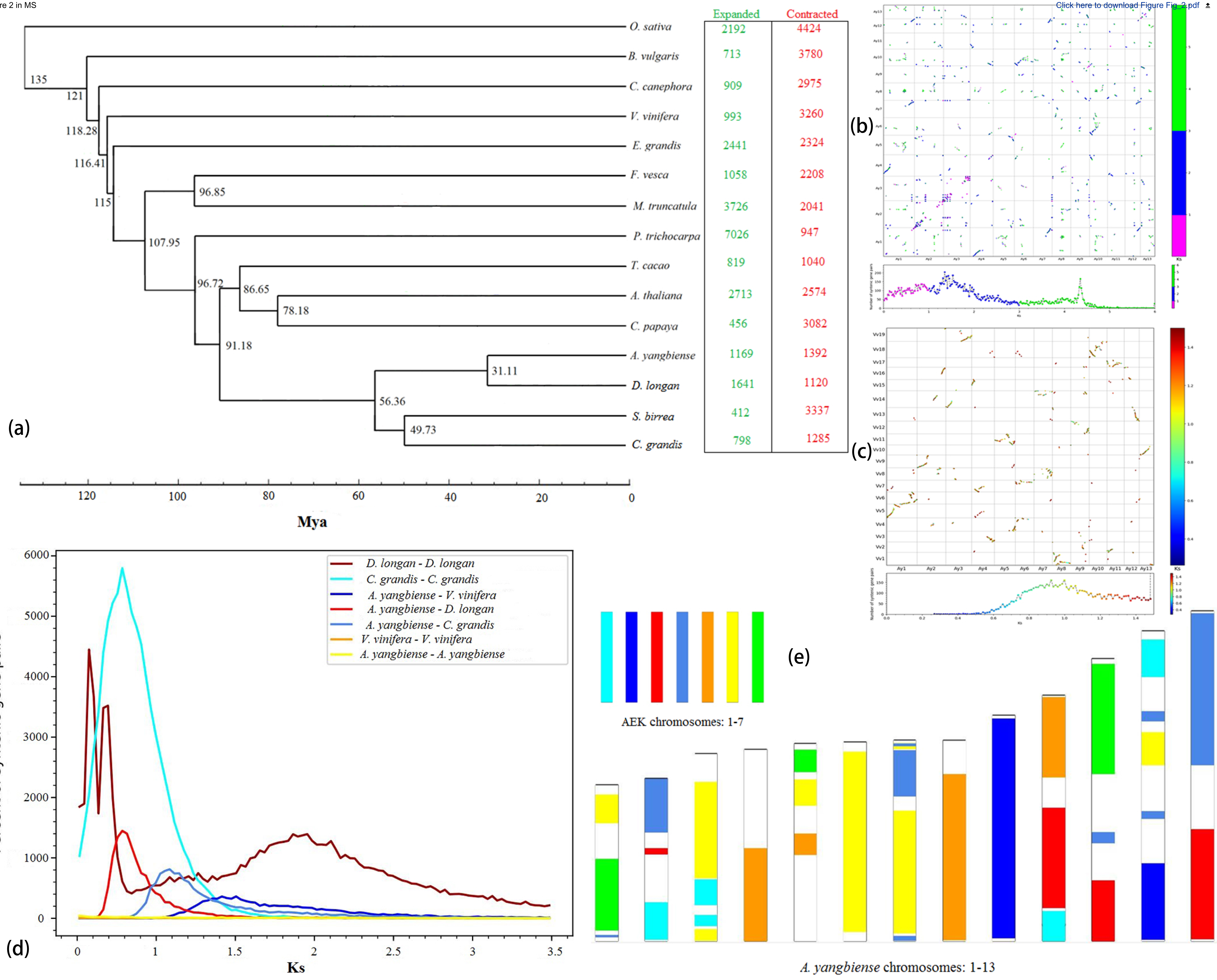

Supplementary Figure 1

Click here to access/download
**Supplementary Material**
Fig_S1.pdf

Supplementary Figure 2

Click here to access/download
**Supplementary Material**
Fig_S2.pdf

Supplementary Figure 3

Click here to access/download
**Supplementary Material**
Fig_S3.pdf

Supplementary Figure 4

Click here to access/download
**Supplementary Material**
Fig_S4.pdf

Supplementary Tables

Click here to access/download
**Supplementary Material**
Supplementary file-Tables.xlsx
