# Supplementary figures and images for: De novo genome assembly of the endangered Acer yangbiense, a plant species with extremely small populations endemic to Yunnan Province, China
Source: Gigascience. 2019 Jul 15;8(7):giz085. doi: 10.1093/gigascience/giz085 (PMC6629541; doi:10.1093/gigascience/giz085)

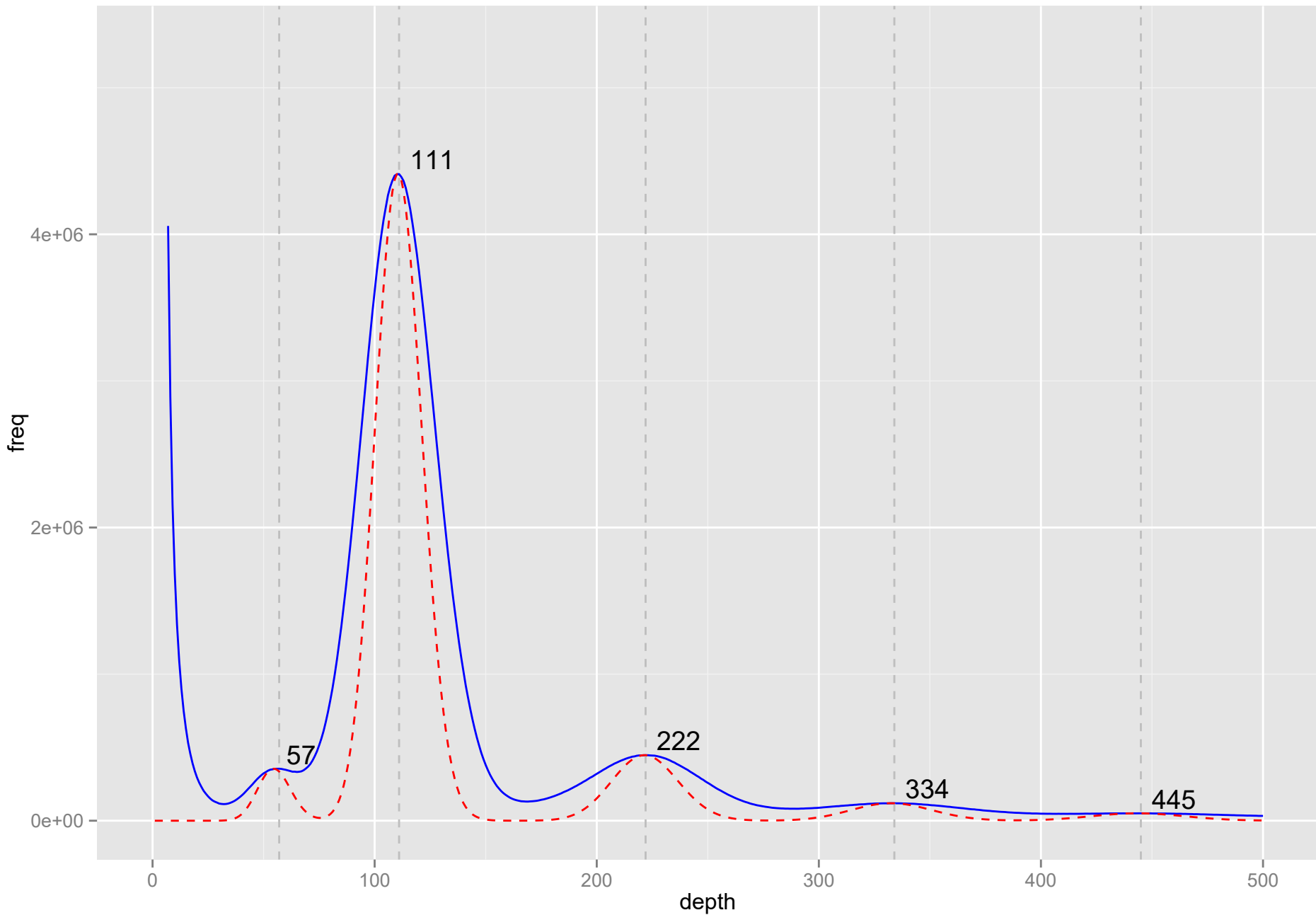

Supplement: giz085_Supplemental_Files [file giz085_supplemental_files.zip › Fig_S1.pdf]

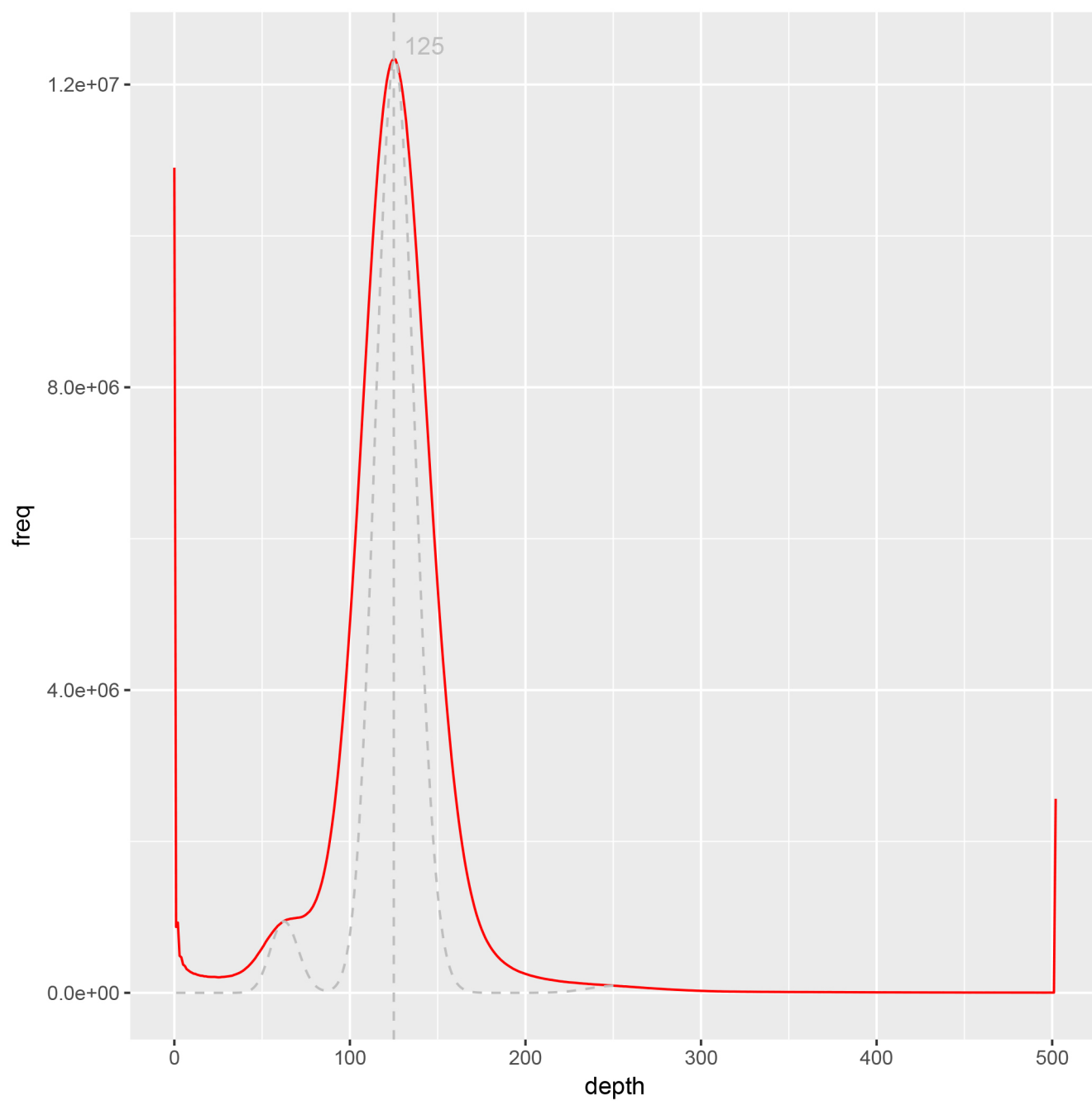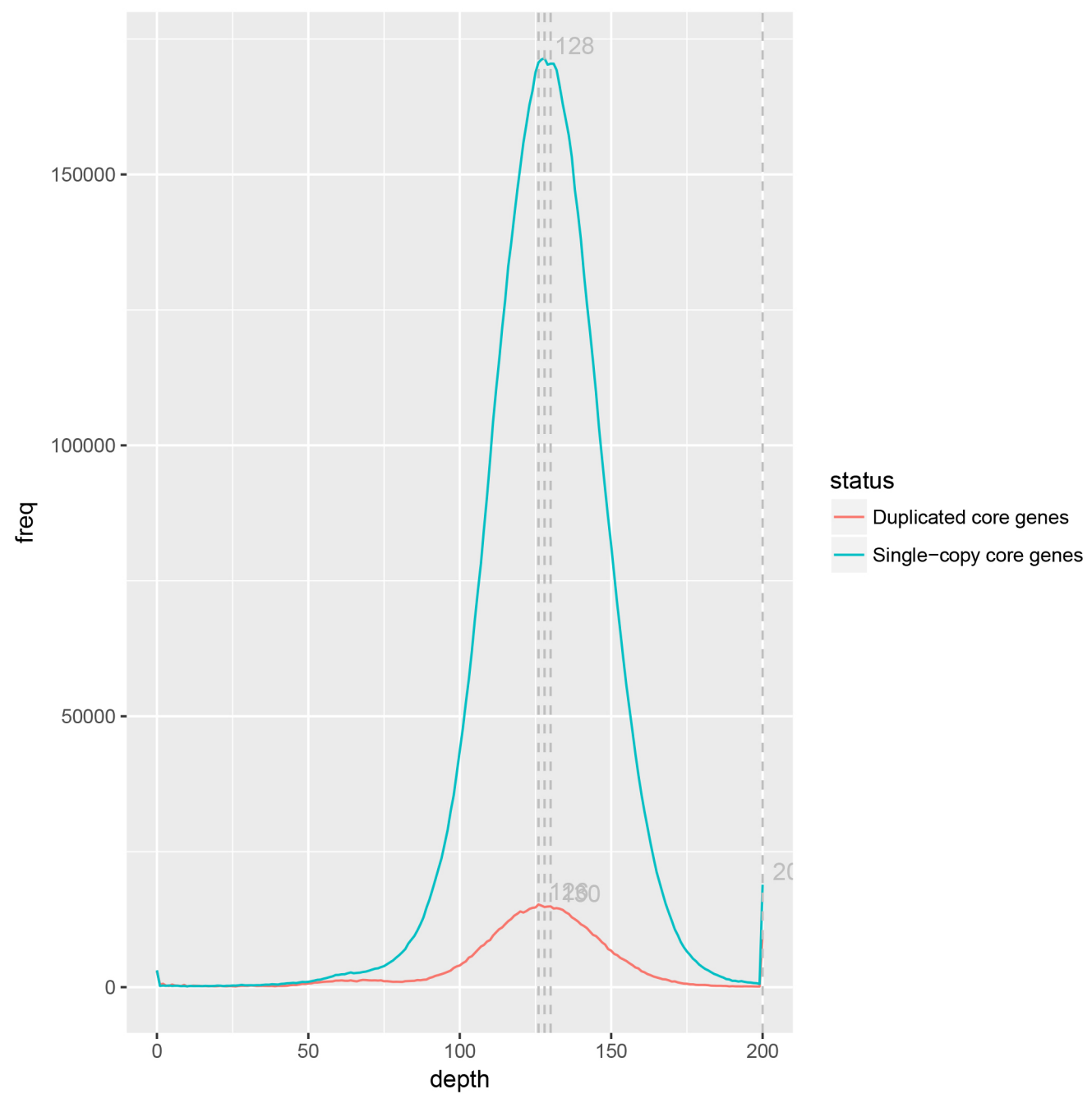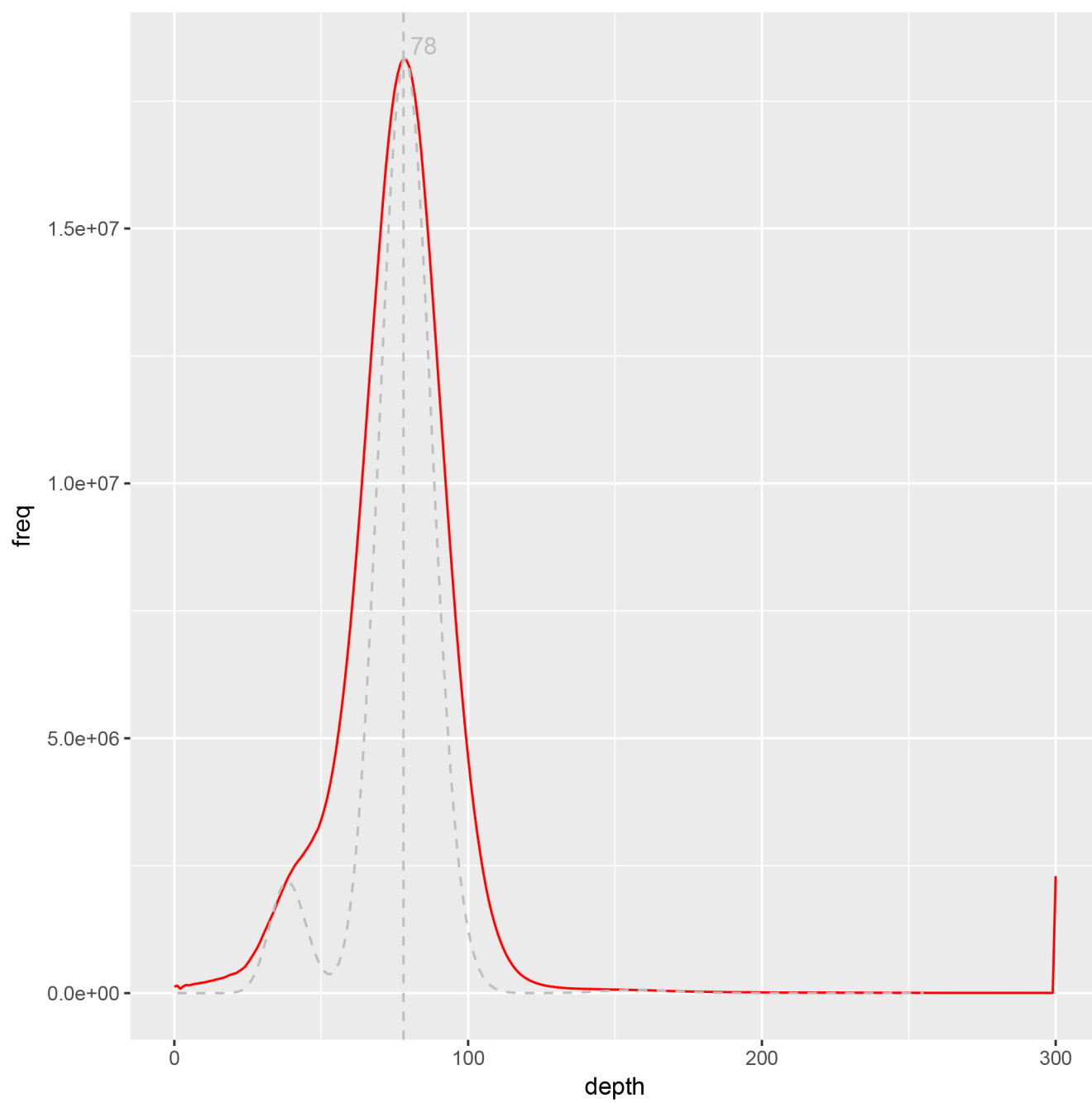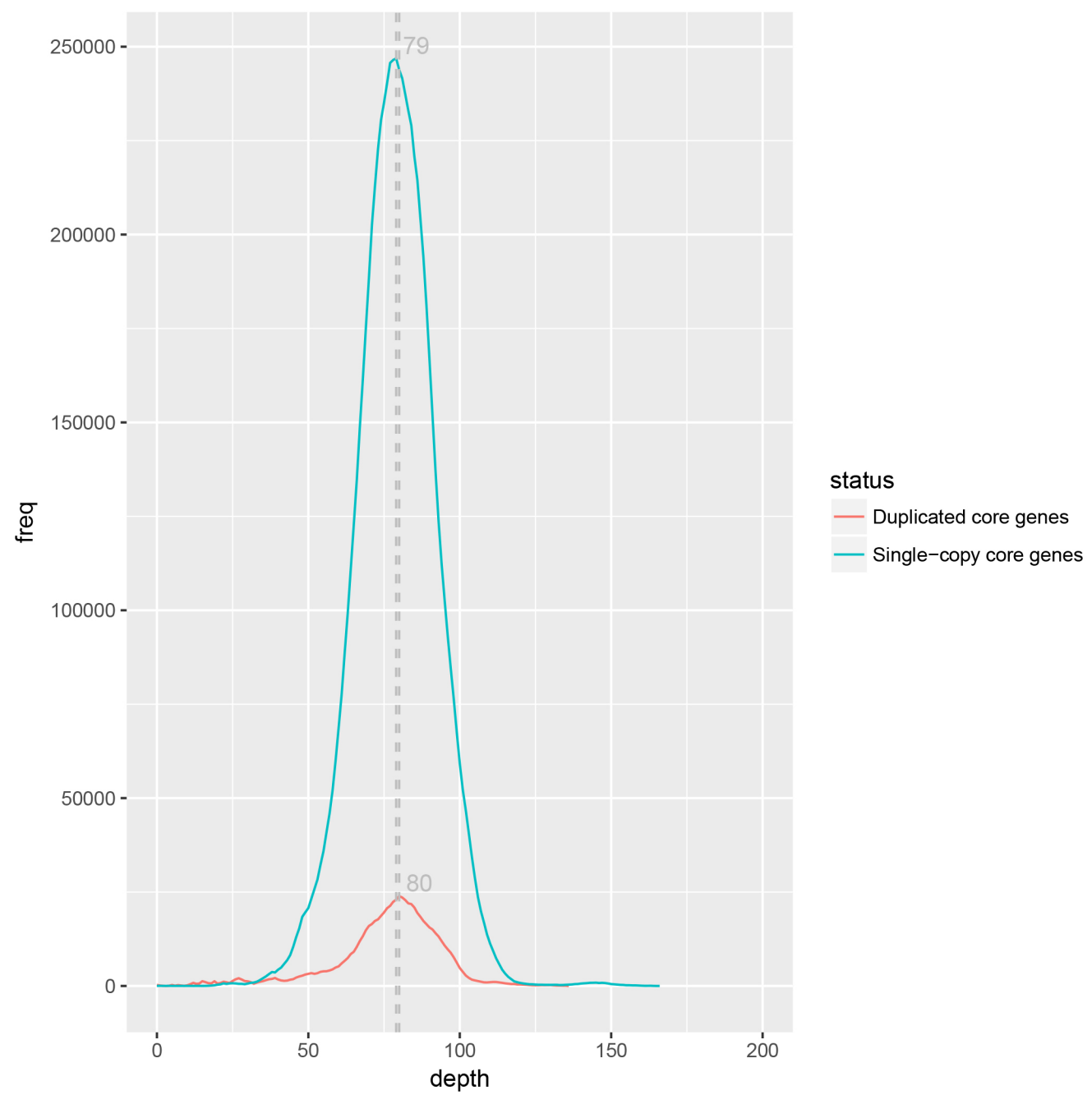

Supplement: giz085_Supplemental_Files [file giz085_supplemental_files.zip › Fig_S2.pdf]

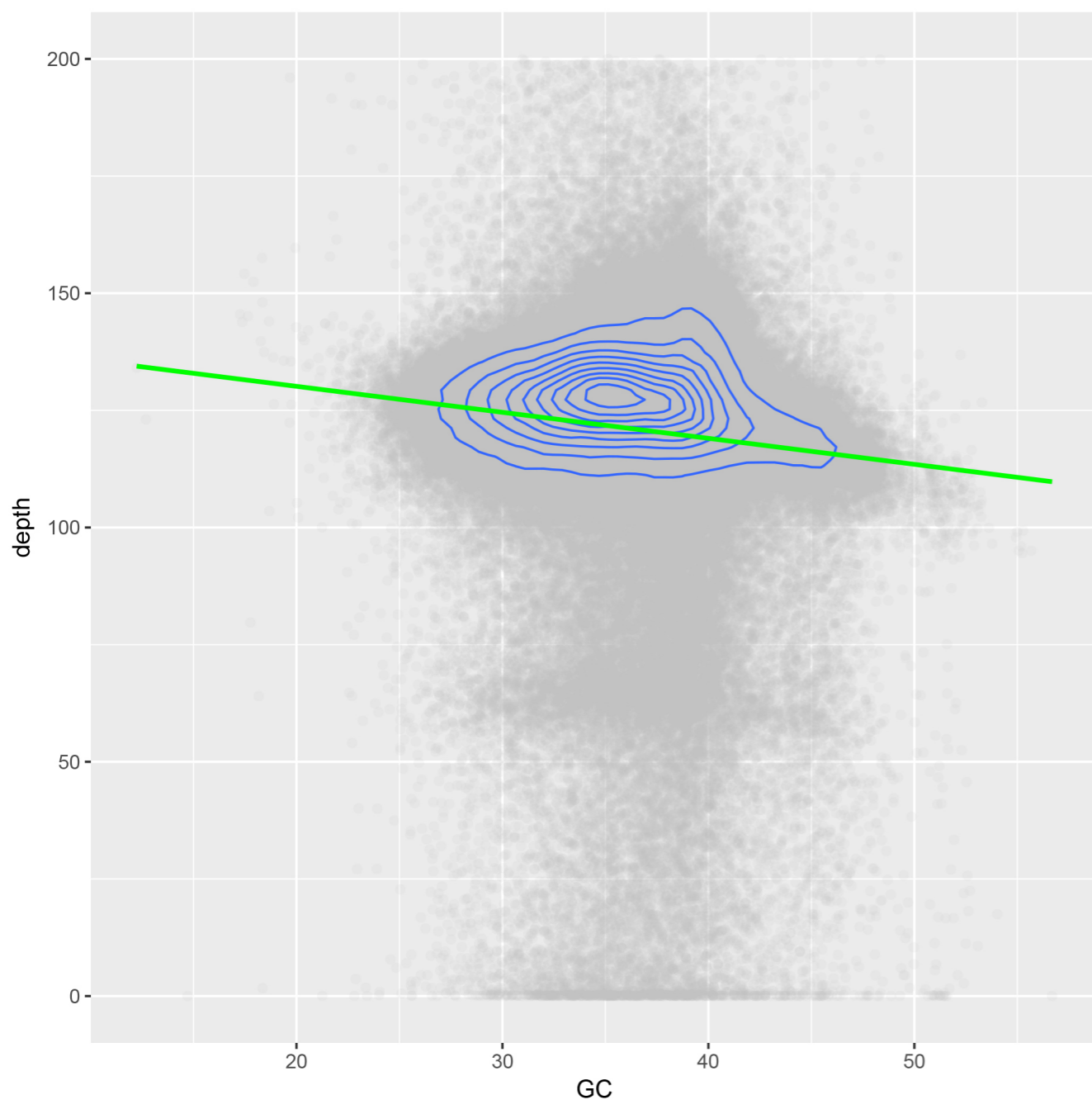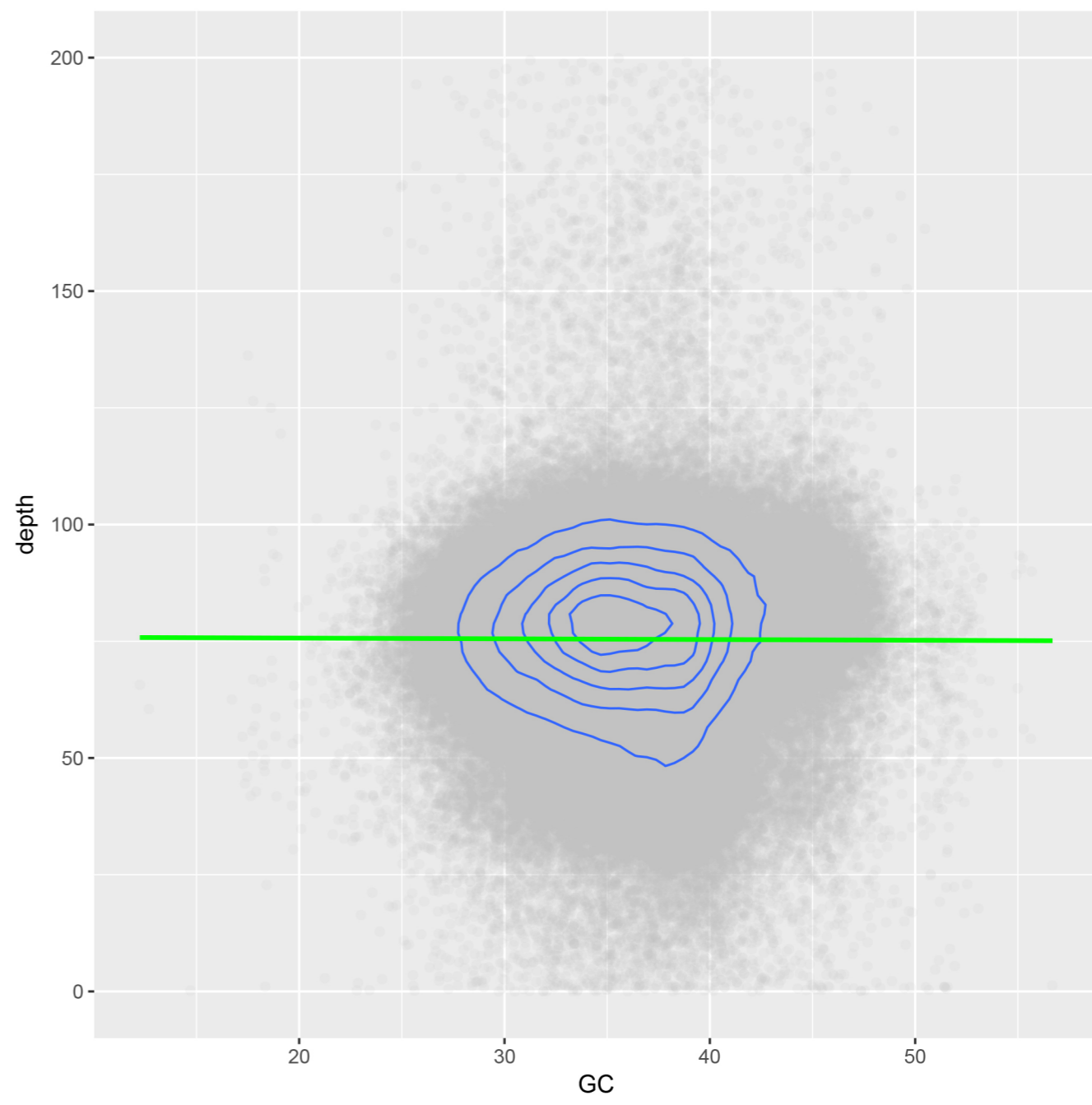

Supplement: giz085_Supplemental_Files [file giz085_supplemental_files.zip › Fig_S3.pdf]

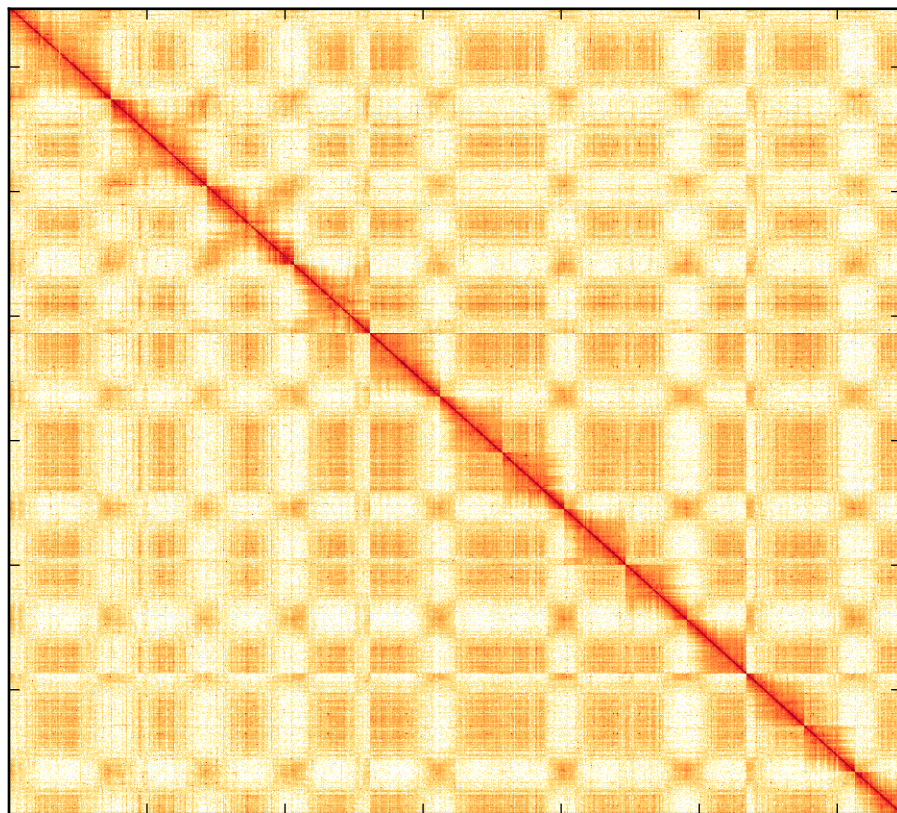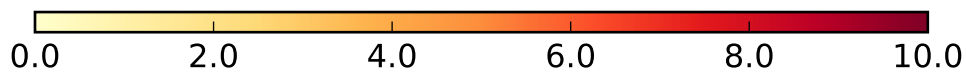

Supplement: giz085_Supplemental_Files [file giz085_supplemental_files.zip › Fig_S4.pdf]
