# Supplementary material for: De novo genome assembly of the endangered Acer yangbiense, a plant species with extremely small populations endemic to Yunnan Province, China
Source: Gigascience. 2019 Jul 15;8(7):giz085. doi: 10.1093/gigascience/giz085 (PMC6629541; doi:10.1093/gigascience/giz085)
Supplement: giz085_Response_to_Reviewer_Comments_Original_Submission [file giz085_response_to_reviewer_comments_original_submission.pdf]

Author's Response To Reviewer Comments

Close

Author's Response to Reviewer Comments

Dear Editor,
Thank you very much for returning our manuscript (GIGA-D-19-00090) entitled
“De novo genome assembly of the endangered Acer yangbiense, a plant species with extremely small populations endemic to Yunnan of China” along with constructive comments and suggestions from three referees. We are very grateful for their efforts and have done our best to respond positively to all comments one by one. However, we have a divergent opinion about the 1st request of reviewer 2 and have explained the reason under it. May the editor pay attention to this one, and we would be appreciated.
We resubmit here the revised manuscript and we hope this version is now suitable for the publication in GigaScience. If you have any further questions or requirements, please do not hesitate to contact the corresponding author.

Yours sincerely,
Yong Peng Ma

Reviewer 1
This study constructed a genome assembly of an endangered plant lives in Yunnan of China. The authors obtained a sufficient amount of short and long NGS reads for the assembly. They then applied Hi-C reads to combine the contigs to scaffolds, and obtained long enough genome sequence that represents the number of chromosomes. The authors applied well-authorized sequencing technologies and assembly strategy and the quality of the obtained sequence seems enough to the downstream analysis. The following studies for gene discovery, annotation, synteny analysis, and evolution estimation are informative and are anticipated to contribute to detail study of this plant species and also with Sapindales. This study is well organized and represent a comprehensive study with sufficient data. The strategy used in this study and the quality is enough for the publication. Thus, I encourage this for publication, but just suggest corrections for those points described below.

1. Cite a paper that describes ploidy and chromosome number of Acer.
Response:
We have added this sentence and its corresponding literature [14] into the first paragraph (Line 47-49) of Background information: The base chromosome number of Acer is x = 13 and the cytological investigation indicate a range of ploidy levels including diploids, tetraploids, hexaploids, octoploids and aneuploids.
[14] Contreras RN, Shearer K. Genome Size, Ploidy, and Base Composition of Wild and Cultivated Acer. Journal of the American Society for Horticultural Science 2018;143(6):470-85.

2. in L180-181; did you applied the Illumina's reads for polymorphism detection with SAMtools?
Response:
Yes, we used Illumina’s reads for polymorphism detection with SAMtools.

3. L189; I suppose Reference 5 in this sentence would be a mistake.
Response:
We are sorry for this mistake, and we have revised it and corrected the reference.

4. in L271-272; reference 94 was skipped.
Response:
In our first manuscript, at the last sentence of previous paragraph, we have cited reference [94]. Before that, [93] has been cited for the first time. That is why in L271-272, it appears that [95] followed [93]. Since we have modified some text and references in this review, it has changed to [95] and [97] (Line

272-273 in this manuscript).

5. Reference 37 and 38; I recommend to append the developer's name.
Response:
We have added the developer’s name into reference number 37 and 38 as suggested by the reviewer.

6. Figure 1a; append a short description (abbreviated code) for each track of the circos plot.
Response:
We have appended abbreviated codes and described the tracks of figure 1a.

7. Figure 1b; describe the meaning of two-to-three letter codes (DHY, MLT, SCH, TP and so on).
Response:
The locations and their codes were described in the legend.

8. Figure 2b,c; describe the Ks plot under each panel briefly.
Response:
We have revised it followed this advice.

9. Figure 2d; the number in vertical scale seems inconsistent to the legend (percent value?).
Response:
The legend of vertical scale in figure 2d should be “Number of syntenic gene pairs”. We made a mistake (“Percent of syntenic gene pairs”) and have revised the legend. Thank you.

Reviewer 2
In this manuscript, Yang et al. present the genome assembly of Acer yangbiense, an endangered maple species found in a restricted geographic region of China. For the most part, the manuscript is well written, and the results presented in the paper will be a useful resource for the community. The assembly appears to be high quality: the sequencing technologies used are recent, the assembly appears to be adequately polished/error-corrected and was chromosome resolved using Hi-C. The repeat masking, transcriptome assembly and annotation with MAKER were all performed in a standard manner. The data (assembly, annotation, and analyses) will be available in GigaDB and raw reads are already publicly available in the SRA.

1. The papers described 7-8 assemblies in the results using a range of genome assemblers (or combinations). I'm not sure how useful this in a data note style paper where the aim is to describe a resource and associated analyses. I would recommend cutting the mentions of trial or pilot assemblies and just describe the method and results for the final assembly (lines 130 - ~ 137)
Response:
We thank for the reviewer’s suggestion and have considered it would be better to save this part in the manuscript for the following reasons: pilot assemblies of earlier versions were corrected and trimmed by different softwares, so that the best assembly was finally polished and chosen. We think that to provide this process, clear idea of how the raw data was analyzed with different ways could be presented to the readers, and makes the methods and the outcomes comparable. However, if the reviewer insists, we are willing to make compromise or let the editor decide if we should save or delete this part.
Here are two references that some of our authors were participated in, in which similar texts were included:
2019. Genome sequence of Malania oleifera, a tree with great value for nervonic acid production. GigaScience 8(2).
2018. High-quality assembly of the reference genome for scarlet sage, Salvia splendens, an economically important ornamental plant. GigaScience 7(7).

2. Line 22 - typo: findings
Response:
We have corrected this typo.

3. Line 154 - 'slightly remove' -> remove putative haplotigs (or something like this)
Response:
We have revised it as suggested by the reviewer.

4. Line 167 - 'map ratio' -> overall alignment rate
Response:
It has been revised as suggested by the reviewer.

5. BLAST versions and names are inconsistent in the manuscript (TblastX -> TBLASTX ; BLAST v2.2.28+ -> BLAST+ v.2.2.28, etc)
Response:
We are sorry for this carelessness. We have followed the instruction and make sure they are in consistent.

Reviewer 3
Minor requests:

1. While Gigascience maintains an ftp site, it is appropriate to upload the actual chromosome sequences and annotation to NCBI or EMBL as well. This is where scientists will look for the genome and be able to use important tools for analysis, such as browse, BLAST, subset, etc.
Response:
Followed this suggestion, we have submitted data of the genome assembly and gene annotation to NCBI. It has passed the initial validation check and will be released to the public database once it is processed. the Accession Number is VAHF00000000 and it is under NCBI BioProject ID PRJNA524417.
The link is:
https://www.ncbi.nlm.nih.gov/bioproject/PRJNA524417
In 'Availability of supporting data' of the manuscript (Line 343-345), we added:
This Whole Genome Shotgun project has been deposited at DDBJ/ENA/GenBank under the accession VAHF00000000. The version described in this paper is version VAHF01000000.

2. Restructure ftp data: The file 'BUSCO_genome.fasta.tgz' unzips into a directory named 'run_genome.fasta'. This is very confusing - only files should be named '.fasta' and its generally accepted that a file with a name ending in .tgz should unzip to that same name (i.e. BUSCO_results.tgz should unzip to a directory named BUSCO_results). Further, this directory contains many files that aren't described in the readme, which they should be if they are to be included.
Response:
We are sorry for it. We reorganized the results of BUSCO and updated the readme.txt, and we have replaced the "BUSCO_genome.fasta.tgz" with "BUSCO_short_summary.txt", which summarized the BUSCO outputs.

3. A few typos:
Abstract
Line 22 - Fingdings to findings
Response:
We have corrected this typo.

4. Methods
Line 22-23 - 'We generated 64 Gb, 94 Gb and 110 Gb of raw DNA sequences' - wouldn't this be 268 Gb? Not clear on why the numbers are reported separately
Response:
We used three different sequencing methodologies and presented their data respectively with the name of these techniques. We have added "respectively" to the end of this sentence and make it clearer: We generated 64 Gb, 94 Gb and 110 Gb of raw DNA sequences and obtained chromosome-level genome assembly of A. yangbiense through a combination of Pacific Biosciences (PacBio) Single-molecule Real-time (SMRT), Illumina HiSeq X, and Hi-C mapping, respectively.

5. RNAMMER and tRNAScan-SE, respectively - mentioned twice
Response:
We have removed one of them.

6. Line 167 - "map ratio" - what is map ratio? Change to 'mapping rate', clarify if unique hits only
Response:
We have modified "map ratio" into mapping rate as suggested by the reviewer.

7. Line 169 - were to was
Response:
We have revised this mistake.

8. Line 169 - 'Illumina short reads and bases' - how are these different datasets?

Line - 'PacBio reads and bases' - same question, I don't understand how this is two different things
Response:
They are different because the calculation of 'reads mapping rate' and 'bases mapping rate' are different. If there are 2 reads, A read is 100 bp, B read is 50 bp. Only A read was mapped, then the 'reads mapping rate' was 50% (1 read was mapped out of 2 reads), while the 'bases mapping rate' would be 66.7% (= 100/150*100%). And for some 'mapped' reads, not all bases of these reads were mapped too. Say maybe A read was mapped, but only 80 bases out of 100 bases of A read were mapped, the 'reads mapping rate' was still 50%, but the 'bases mapping rate' would be 53.3% (= 80/150*100%).
In our results: PacBio sequencing produced reads with an average read length of 10Kb, and Illumina sequencing produced reads with average read length of 148.5 bp. The reads length varied much from two sequencing methods. For Illumina, short length of reads makes mapping rate of reads or bases similar, we have deleted base mapping rate in the manuscript. But for PacBio SMRT sequencing, the value of 'bases mapping rate' would be higher than 'reads mapping rate', because many short reads couldn't map, but higher proportions of bases in long reads mapped.

9. Line 181 - were to was
Response:
We have revised this mistake.

10. Line 250 - 'As a result, 26,257 (92.7%) and 27,827 (98.26%) protein-coding genes out of the total 28,320 genes in the A. yangbiense genome were assigned potential functions by BLAT and InterProScan, respectively' - This doesn't seem likely - even Arabidopsis, the best annotated plant genome still has many "hypothetical" and "unknown" function genes.
Response:
We thank the reviewer for discovering this improper narrative. We have replaced this sentence with: The highest proportions of annotation were 92.60% (database NR) by BLAT and 92.31% (database PANTHER) by InterProScan and the unannotated proportion were 7.30% and 1.74, respectively (Supplementary Table S10).

11. Figure 1 - misspelling of chromosomes in legend
Change track to tracks
Response:
We have revised the typos, and the legend of figure 1a was rewritten.

12. "functional categories" - remove function, only one of the categories deals with function
Response:
We have removed the word 'functional' when referring to categories as suggested.

Close
