# Supplementary material for: De novo genome assembly of the endangered Acer yangbiense, a plant species with extremely small populations endemic to Yunnan Province, China
Source: Gigascience. 2019 Jul 15;8(7):giz085. doi: 10.1093/gigascience/giz085 (PMC6629541; doi:10.1093/gigascience/giz085)
Supplement: giz085_Reviewer_1_Report_Original_Submission — Tokurou Shimizu -- 4/20/2019 Reviewed [file giz085_reviewer_1_report_original_submission.pdf]

Reviewer Report

Title: De novo genome assembly of the endangered Acer yangbiense, a plant species with extremely small populations endemic to Yunnan of China

Version: Original Submission **Date:** 4/20/2019

Reviewer name: Tokurou Shimizu

Reviewer Comments to Author:

This study constructed a genome assembly of an endangered plant lives in Yunnan of China.
The authors obtained a sufficient amount of short and long NGS reads for the assembly. They then applied Hi-C reads to combine the contigs to scaffolds, and obtained long enough genome sequence that represents the number of chromosomes. The authors applied well-authorized sequencing technologies and assembly strategy and the quality of the obtained sequence seems enough to the downstream analysis.
The following studies for gene discovery, annotation, synteny analysis, and evolution estimation are informative and are anticipated to contribute to detail study of this plant species and also with Sapindales.
This study is well organized and represent a comprehensive study with sufficient data. The strategy used in this study and the quality is enough for the publication.
Thus, I encourage this for publication, but just suggest corrections for those points described below.
1. Cite a paper that describes ploidy and chromosome number of Acer.
2. in L180-181; did you applied the Illumina's reads for polymorphism detection with SAMtools?
3. L189; I suppose Reference 5 in this sentence would be a mistake.
4. in L271-272; reference 94 was skipped.
5. Reference 37 and 38; I recommend to append the developer's name.
6. Figure 1a; append a short description (abbreviated code) for each track of the circos plot.
7. Figure 1b; describe the meaning of two-to-three letter codes (DHY, MLT, SCH, TP and so on).
8. Figure 2b,c; describe the Ks plot under each panel briefly.
9. Figure 2d; the number in vertical scale seems inconsistent to the legend (percent value?).

Level of Interest

Please indicate how interesting you found the manuscript: Choose an item.

Quality of Written English

Please indicate the quality of language in the manuscript: Choose an item.

Declaration of Competing Interests

Please complete a declaration of competing interests, considering the following questions:

- Have you in the past five years received reimbursements, fees, funding, or salary from an organisation that may in any way gain or lose financially from the publication of this manuscript, either now or in the future?
- Do you hold any stocks or shares in an organisation that may in any way gain or lose financially from the publication of this manuscript, either now or in the future?
- Do you hold or are you currently applying for any patents relating to the content of the manuscript?
- Have you received reimbursements, fees, funding, or salary from an organization that holds or has applied for patents relating to the content of the manuscript?
- Do you have any other financial competing interests?
- Do you have any non-financial competing interests in relation to this paper?

If you can answer no to all of the above, write 'I declare that I have no competing interests' below. If your reply is yes to any, please give details below.

I declare that I have no competing interests.

I agree to the open peer review policy of the journal. I understand that my name will be included on my report to the authors and, if the manuscript is accepted for publication, my named report including any attachments I upload will be posted on the website along with the authors' responses. I agree for my report to be made available under an Open Access Creative Commons CC-BY license (http://creativecommons.org/licenses/by/4.0/). I understand that any comments which I do not wish to be included in my named report can be included as confidential comments to the editors, which will not be published.

I agree to the open peer review policy of the journal

To further support our reviewers, we have joined with Publons, where you can gain additional credit to further highlight your hard work (see: https://publons.com/journal/530/gigascience). On publication of this paper, your review will be automatically added to Publons, you can then choose whether or not to claim your Publons credit. I understand this statement.

Yes Choose an item.
