# Supplementary material for: De novo genome assembly of the endangered Acer yangbiense, a plant species with extremely small populations endemic to Yunnan Province, China
Source: Gigascience. 2019 Jul 15;8(7):giz085. doi: 10.1093/gigascience/giz085 (PMC6629541; doi:10.1093/gigascience/giz085)
Supplement: giz085_Reviewer_2_Report_Original_Submission — John Hamilton -- 4/24/2019 Reviewed [file giz085_reviewer_2_report_original_submission.pdf]

**Reviewer Report**

**Title: De novo genome assembly of the endangered Acer yangbiense, a plant species with extremely small populations endemic to Yunnan of China**

**Version: Original Submission** **Date:** 4/24/2019

**Reviewer name: John Hamilton**

**Reviewer Comments to Author:**

In this manuscript, Yang et al. present the genome assembly of Acer yangbiense, an endangered maple species found in a restricted geographic region of China. For the most part, the manuscript is well written, and the results presented in the paper will be a useful resource for the community. The assembly appears to be high quality: the sequencing technologies used are recent, the assembly appears to be adequately polished/error-corrected and was chromosome resolved using Hi-C. The repeat masking, transcriptome assembly and annotation with MAKER were all performed in a standard manner. The data (assembly, annotation, and analyses) will be available in GigaDB and raw reads are already publicly available in the SRA.

Comments:

1) The papers described 7-8 assemblies in the results using a range of genome assemblers (or combinations). I'm not sure how useful this in a data note style paper where the aim is to describe a resource and associated analyses. I would recommend cutting the mentions of trial or pilot assemblies and just describe the method and results for the final assembly (lines 130 - ~ 137)

2) Line 22 - typo: findings

3) Line 154 - 'slightly remove' -> remove putative haplotigs (or something like this)

4) Line 167 - 'map ratio' -> overall alignment rate

5) BLAST versions and names are inconsistent in the manuscript ( TblastX -> TBLASTX ; BLAST v2.2.28+ -> BLAST+ v.2.2.28, etc)

**Level of Interest**

Please indicate how interesting you found the manuscript: Choose an item.

**Quality of Written English**

Please indicate the quality of language in the manuscript: Choose an item.

**Declaration of Competing Interests**

Please complete a declaration of competing interests, considering the following questions:

- Have you in the past five years received reimbursements, fees, funding, or salary from an organisation that may in any way gain or lose financially from the publication of this manuscript, either now or in the future?
- Do you hold any stocks or shares in an organisation that may in any way gain or lose financially from the publication of this manuscript, either now or in the future?
- Do you hold or are you currently applying for any patents relating to the content of the manuscript?
- Have you received reimbursements, fees, funding, or salary from an organization that holds or has applied for patents relating to the content of the manuscript?
- Do you have any other financial competing interests?
- Do you have any non-financial competing interests in relation to this paper?

If you can answer no to all of the above, write 'I declare that I have no competing interests' below. If your reply is yes to any, please give details below.

I declare that I have no competing interests

I agree to the open peer review policy of the journal. I understand that my name will be included on my report to the authors and, if the manuscript is accepted for publication, my named report including any attachments I upload will be posted on the website along with the authors' responses. I agree for my report to be made available under an Open Access Creative Commons CC-BY license (http://creativecommons.org/licenses/by/4.0/). I understand that any comments which I do not wish to be included in my named report can be included as confidential comments to the editors, which will not be published.

I agree to the open peer review policy of the journal

To further support our reviewers, we have joined with Publons, where you can gain additional credit to further highlight your hard work (see: https://publons.com/journal/530/gigascience). On publication of this paper, your review will be automatically added to Publons, you can then choose whether or not to claim your Publons credit. I understand this statement.

Yes Choose an item.
