# Supplementary material for: De novo genome assembly of the endangered Acer yangbiense, a plant species with extremely small populations endemic to Yunnan Province, China
Source: Gigascience. 2019 Jul 15;8(7):giz085. doi: 10.1093/gigascience/giz085 (PMC6629541; doi:10.1093/gigascience/giz085)
Supplement: giz085_Reviewer_3_Report_Original_Submission — Meg Staton -- 4/29/2019 Reviewed [file giz085_reviewer_3_report_original_submission.pdf]

Reviewer Report

Title: De novo genome assembly of the endangered Acer yangbiense, a plant species with extremely small populations endemic to Yunnan of China

Version: Original Submission **Date:** 4/29/2019

Reviewer name: Meg Staton

Reviewer Comments to Author:

Minor requests:
- While Gigascience maintains an ftp site, it is appropriate to upload the actual chromosome sequences and annotation to NCBI or EMBL as well. This is where scientists will look for the genome and be able to use important tools for analysis, such as browse, BLAST, subset, etc.
- Restructure ftp data: The file 'BUSCO_genome.fasta.tgz' unzips into a directory named 'run_genome.fasta'. This is very confusing - only files should be named '.fasta' and its generally accepted that a file with a name ending in .tgz should unzip to that same name (i.e. BUSCO_results.tgz should unzip to a directory named BUSCO_results). Further, this directory contains many files that aren't described in the readme, which they should be if they are to be included.

A few typos:
Abstract
Line 22 - Fingdings to findings
Methods
Line 22-23 - 'We generated 64 Gb, 94 Gb and 110 Gb of raw DNA sequences' - wouldn't this be 268 Gb? Not clear on why the numbers are reported separately
RNAMMER and tRNAScan-SE, respectively - mentioned twice
Lilne 167 - "map ratio" - what is map ratio? Change to 'mapping rate', clarify if unique hits only
Linr 169 - were to was
Line 169 - 'Illumina short reads and bases' - how are these different datasets?
Line - 'PacBio reads and bases' - same question, I don't undertand how this is two different things
Line 181 - were to was
Line 250 - 'As a result, 26,257 (92.7%) and 27,827 (98.26%) protein-coding genes out of the total 28,320 genes in the A. yangbiense genome were assigned potential functions by BLAT and InterProScan, respectively' - This doesn't seem likely - even Arabidopsis, the best annotated plant genome still has many "hypothetical" and "unknown" function genes.
Figure 1 - misspelling of chromosomes in legend
Change track to tracks
"functional categories" - remove function, only one of the categories deals with function

Level of Interest

Please indicate how interesting you found the manuscript: Choose an item.

Quality of Written English

Please indicate the quality of language in the manuscript: Choose an item.

Declaration of Competing Interests

Please complete a declaration of competing interests, considering the following questions:

- Have you in the past five years received reimbursements, fees, funding, or salary from an organisation that may in any way gain or lose financially from the publication of this manuscript, either now or in the future?
- Do you hold any stocks or shares in an organisation that may in any way gain or lose financially from the publication of this manuscript, either now or in the future?
- Do you hold or are you currently applying for any patents relating to the content of the manuscript?
- Have you received reimbursements, fees, funding, or salary from an organization that holds or has applied for patents relating to the content of the manuscript?
- Do you have any other financial competing interests?
- Do you have any non-financial competing interests in relation to this paper?

If you can answer no to all of the above, write 'I declare that I have no competing interests' below. If your reply is yes to any, please give details below.

I declare that I have no competing interests.

I agree to the open peer review policy of the journal. I understand that my name will be included on my report to the authors and, if the manuscript is accepted for publication, my named report including any attachments I upload will be posted on the website along with the authors' responses. I agree for my report to be made available under an Open Access Creative Commons CC-BY license (http://creativecommons.org/licenses/by/4.0/). I understand that any comments which I do not wish to be included in my named report can be included as confidential comments to the editors, which will not be published.

I agree to the open peer review policy of the journal

To further support our reviewers, we have joined with Publons, where you can gain additional credit to further highlight your hard work (see: https://publons.com/journal/530/gigascience). On publication of this paper, your review will be automatically added to Publons, you can then choose whether or not to claim your Publons credit. I understand this statement.

Yes Choose an item.
